# Supplementary figures and images for: Combinatorial Wnt signaling landscape during brachiopod anteroposterior patterning
Source: BMC Biol. 2024 Sep 19;22:212. doi: 10.1186/s12915-024-01988-w (PMC11414264; doi:10.1186/s12915-024-01988-w)

[illegible]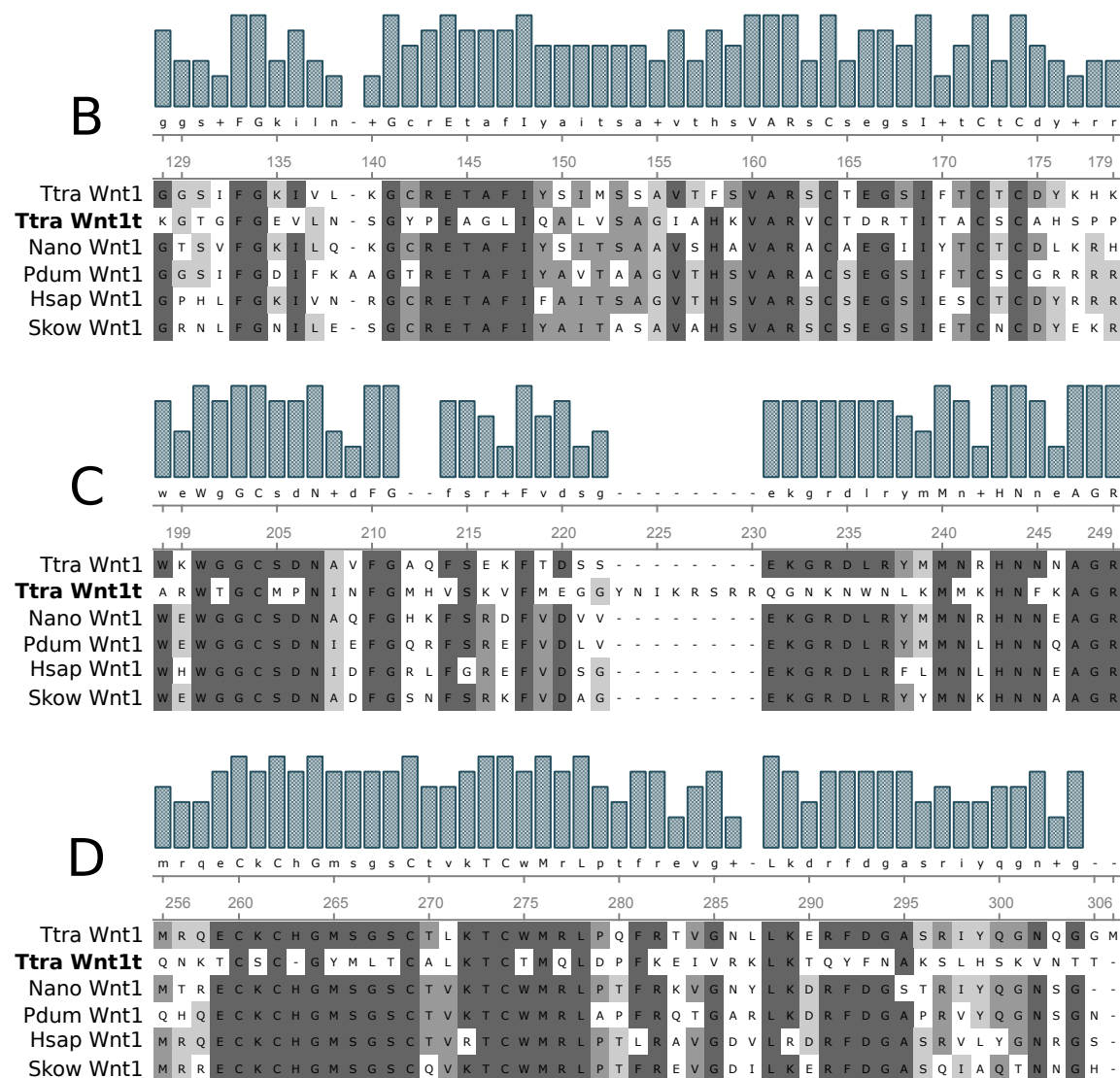

Supplement: Supplementary file 1 — Additional file 1: Fig. S1: [PDF] Domain architecture of Terebratalia transversa Wnt proteins. A Schematic drawings showing signal peptide regions, Wnt protein signatures, Frizzled-receptor binding sites, and C-terminal Wnt domain based on InterProScan annotations. All T. transversa have a similar overall architecture. B–C Multiple sequence alignment of Wnt1 proteins, showing the highly divergent sequence of T. transversa Wnt1t in three Wnt protein signature regions. The alignment contains Wnt1 orthologs of T. transversa (Ttra), Novocrania anomala (Nano), Platynereis dumerilii (Pdum), Homo sapiens (Hsap), and Saccoglossus kowalevskii (Skow). [file 12915_2024_1988_MOESM1_ESM.pdf]

Tree scale: 0.1

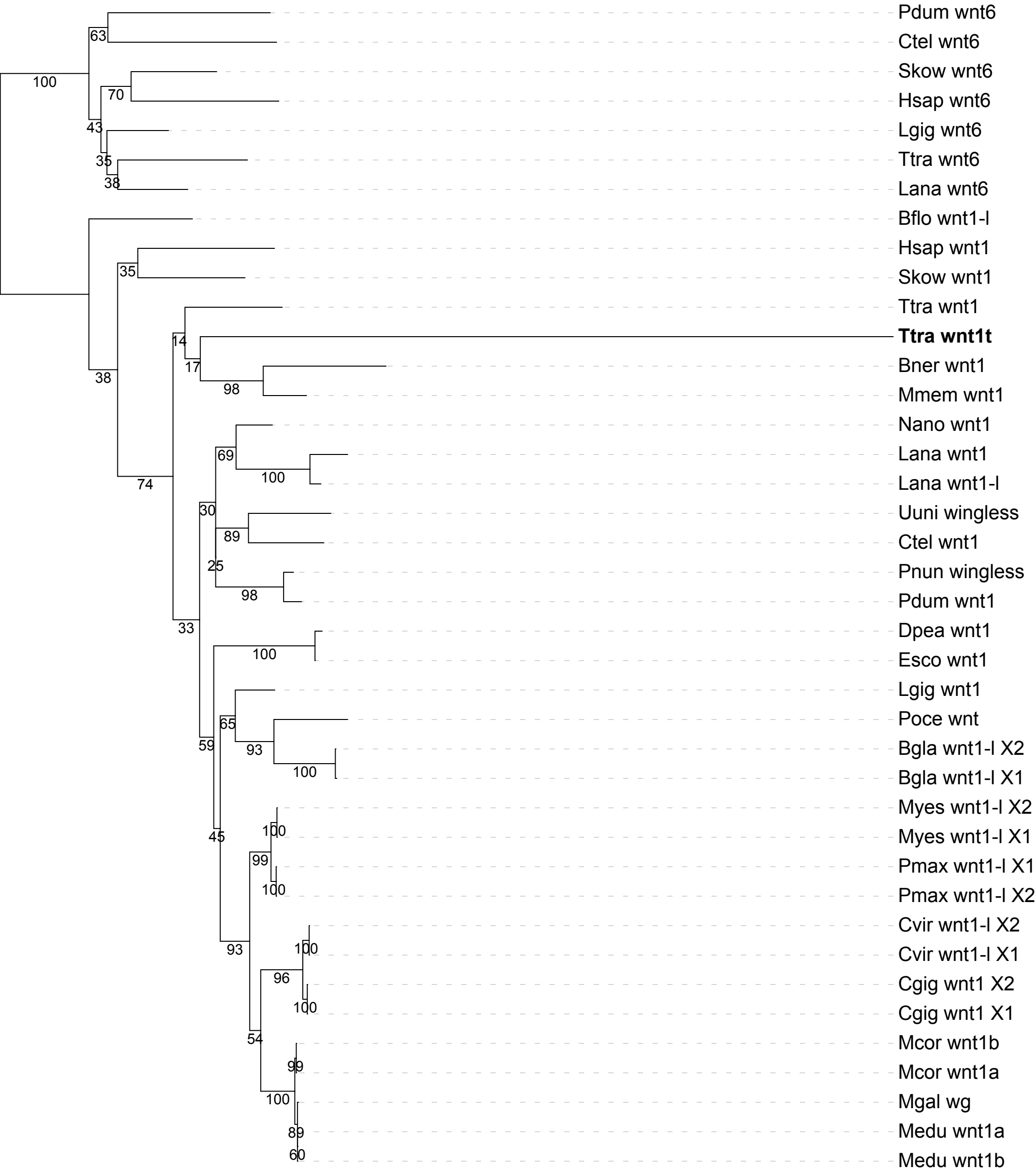

Supplement: Supplementary file 2 — Additional file 2: Fig. S2: [PDF] Phylogenetic analysis of Terebratalia transversa Wnt1 proteins. Best-scoring tree of a maximum likelihood phylogenetic analysis using amino acid sequences of genes from the wnt1 subfamily with wnt6 as an outgroup. Branch lengths are proportional to the amount of sequence change, and the numbers show the support values of individual branches. Both Terebratalia transversa (Ttra) and Lingula anatina (Lana), a rhynchonelliform and a linguliform brachiopod, respectively, have two copies of wnt1. If this was an ancient duplication event at the base of Brachiopoda, we would expect the orthologous wnt1 paralogs from different species to cluster together (i.e., Ttra wnt1 with Lana wnt1). Instead, the tree reveals that the paralog copies of each species cluster together, suggesting that the duplication of wnt1 occurred independently in T. transversa and L. anatina. T. transversa wnt1t also shows a longer branch length indicating rapid evolution. Taxon sampling was focused in spiralians. The other species are Biomphalaria glabrata (Bgla), Branchiostoma floridae (Bflo), Bugula neritina (Bner), Capitella teleta (Ctel), Crassostrea virginica (Cvir), Doryteuthis pealeii (Dpea), Euprymna scolopes (Esco), Homo sapiens (Hsap), Lingula anatina (Lana), Lottia gigantea (Lgig), Membranipora membranacea (Mmem), Mizuhopecten yessoensis (Myes), Mytilus coruscus (Mcor), Mytilus edulis (Medu), Mytilus galloprovincialis (Mgal), Pecten maximus (Pmax), Perinereis nuntia (Pnun), Plakobranchus ocellatus (Poce), Platynereis dumerilii (Pdum), Saccoglossus kowalevskii (Skow), and Urechis unicinctus (Uuni). [file 12915_2024_1988_MOESM2_ESM.pdf]

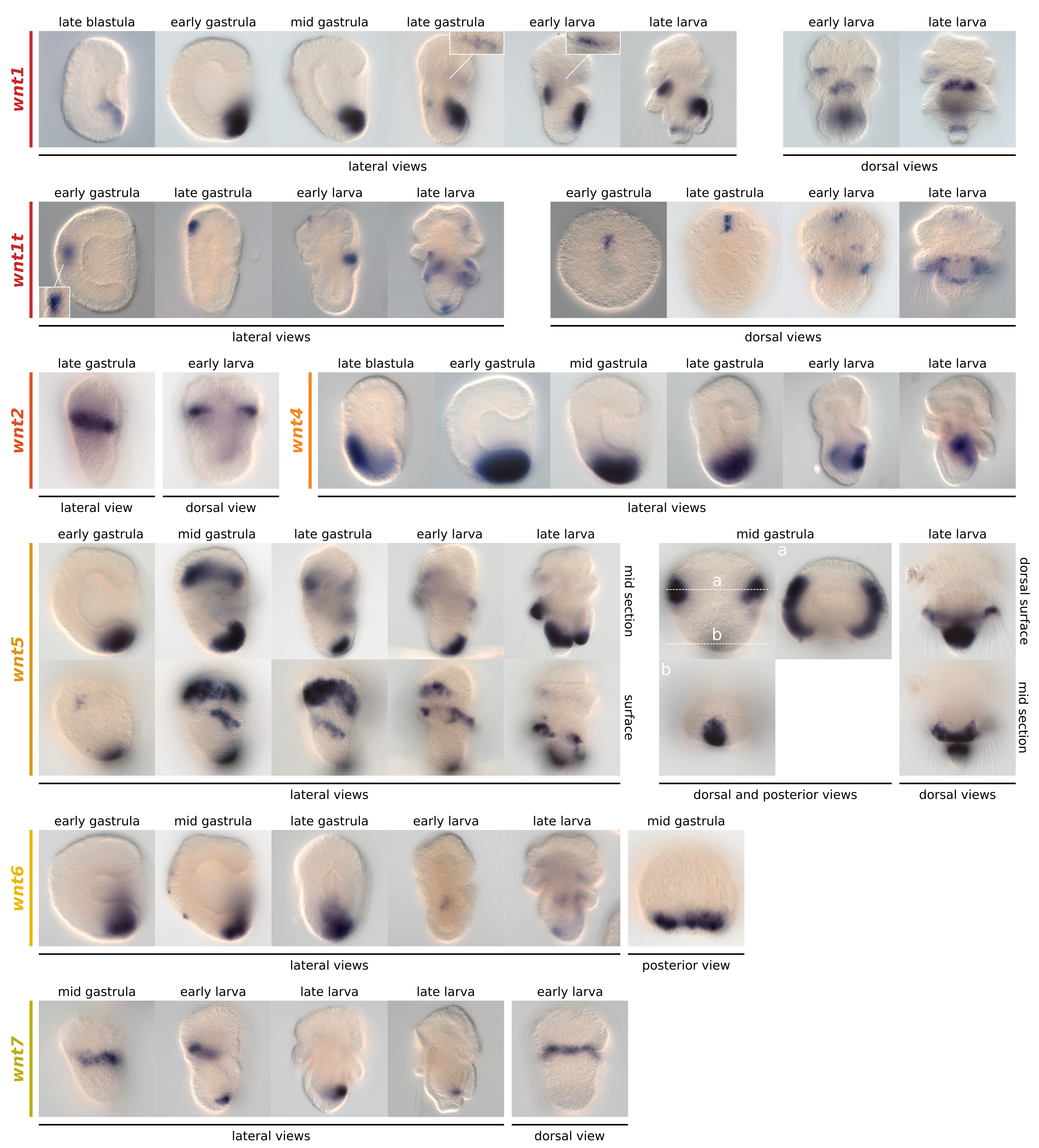

Supplement: Supplementary file 3 — Additional file 3: Fig. S3: [PNG] Whole-mount colorimetric in situ hybridization of wnt1, wnt1t, wnt2, wnt4, wnt5, wnt6, and wnt7 in Terebratalia transversa. Additional views of Wnt expression between late blastula and late larva. Dashed lines indicate the position of the optical section shown in adjacent panels. The panels show representative expression patterns for each sample. [file 12915_2024_1988_MOESM3_ESM.png]

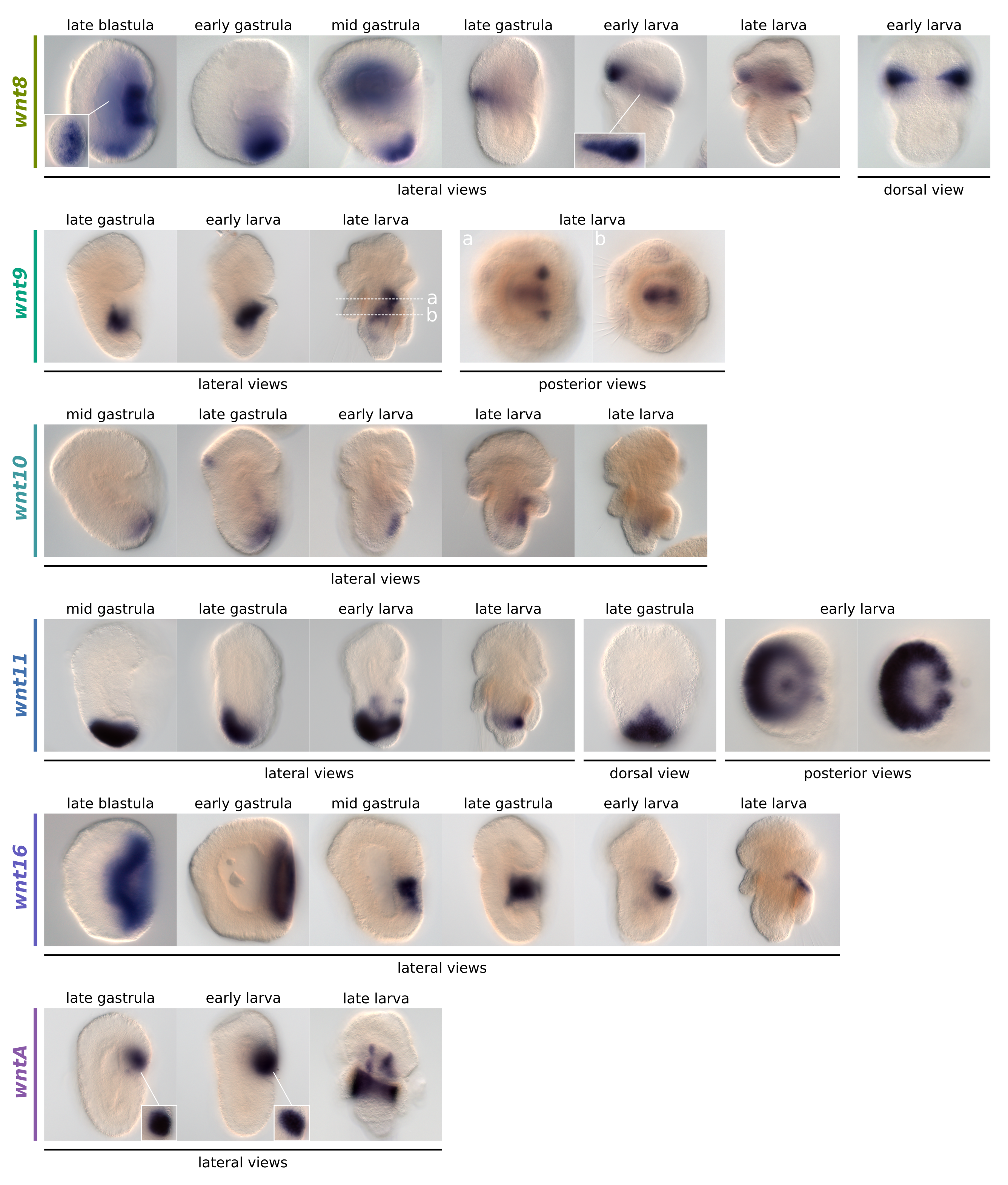

Supplement: Supplementary file 4 — Additional file 4: Fig. S4: [PNG] Whole-mount colorimetric in situ hybridization of wnt8, wnt9, wnt10, wnt11, wnt16, and wntA in Terebratalia transversa. Additional views of Wnt expression between late blastula and late larva. Dashed lines indicate the position of the optical section shown in adjacent panels. The panels show representative expression patterns for each sample. [file 12915_2024_1988_MOESM4_ESM.png]

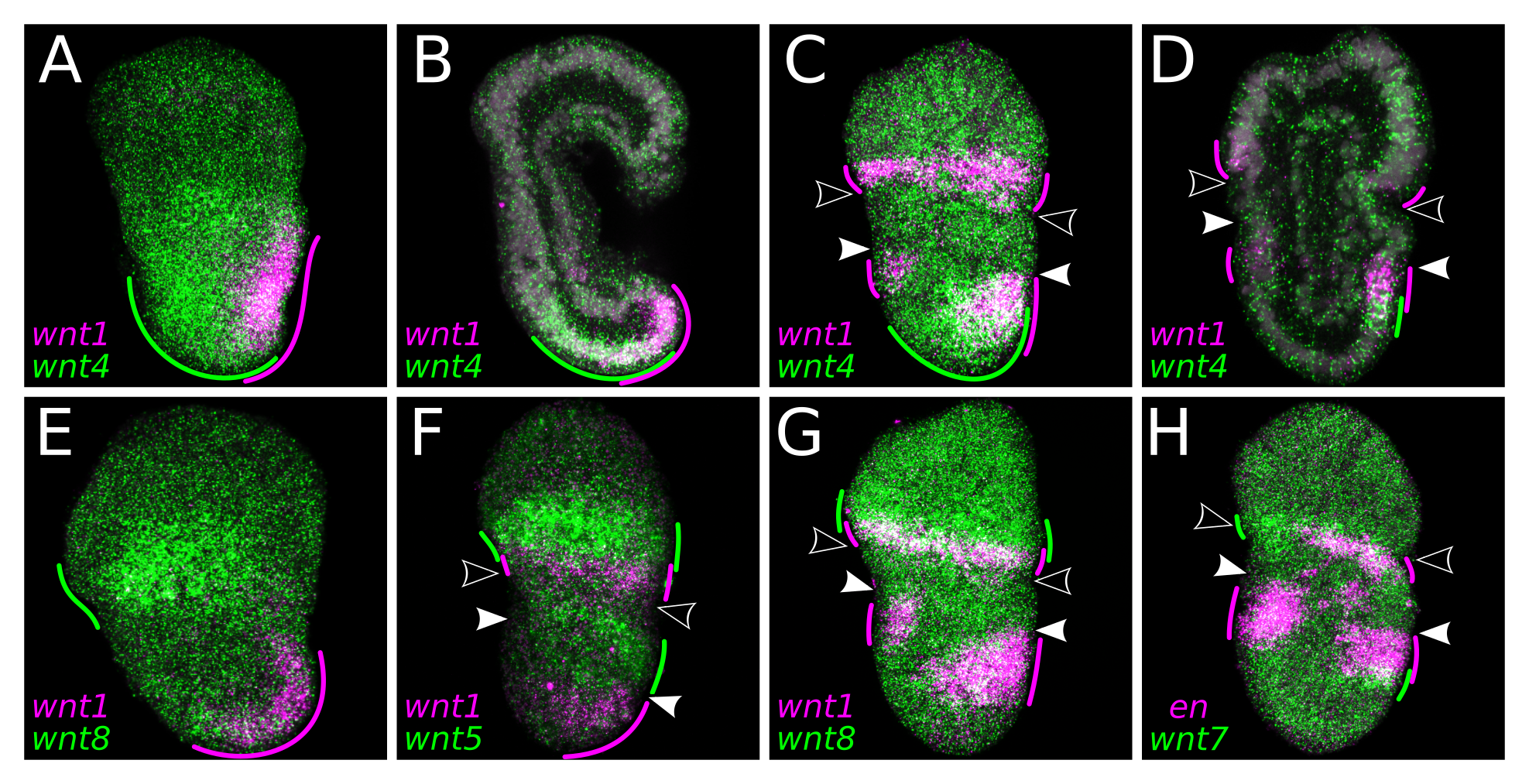

Supplement: Supplementary file 5 — Additional file 5: Fig. S5: [PNG] Whole-mount double-fluorescent in situ hybridization of Terebratalia transversa wnt genes. A–D Expression of wnt1 (magenta) and wnt4 (green) in the mid gastrula (A,B) and late gastrula (C,D). E,G Expression of wnt1 (magenta) and wnt8 (green) in the mid gastrula (E) and early larva (G). F Expression of wnt1 (magenta) and wnt5 (green) in the late gastrula. H Expression of engrailed (magenta) and wnt7 (green) in the early larva. Green and magenta lines highlight the extension and overlap between domains. Areas in the tissue where the expression overlaps appear in white. Samples oriented with anterior end to the top and ventral to the right (lateral views). Black arrowheads indicate the apical–mantle boundary. White arrowheads demarcate the mantle–pedicle boundary. The panels show representative expression patterns for each sample. [file 12915_2024_1988_MOESM5_ESM.png]

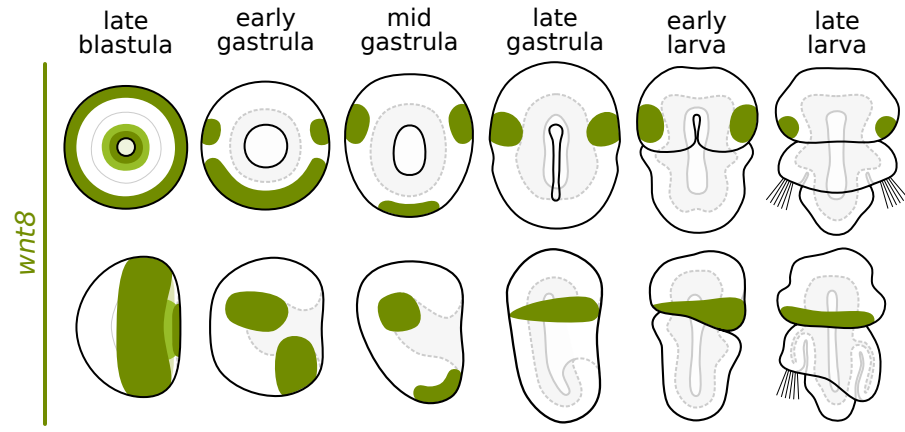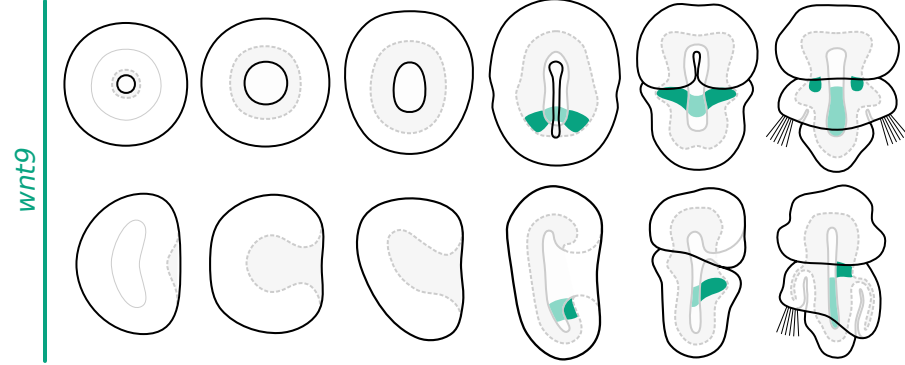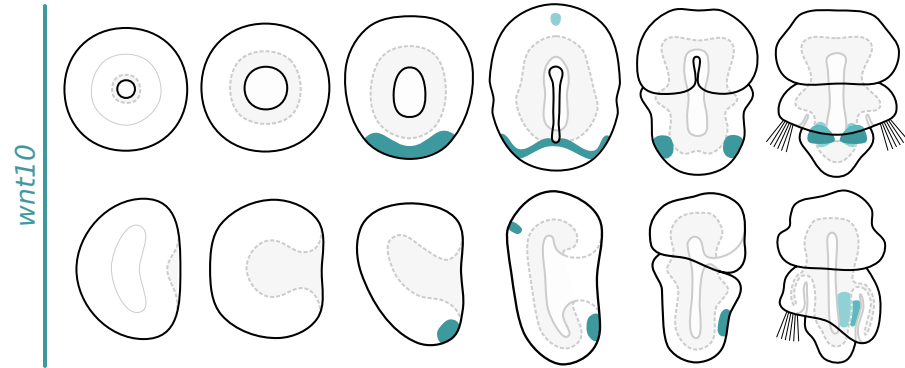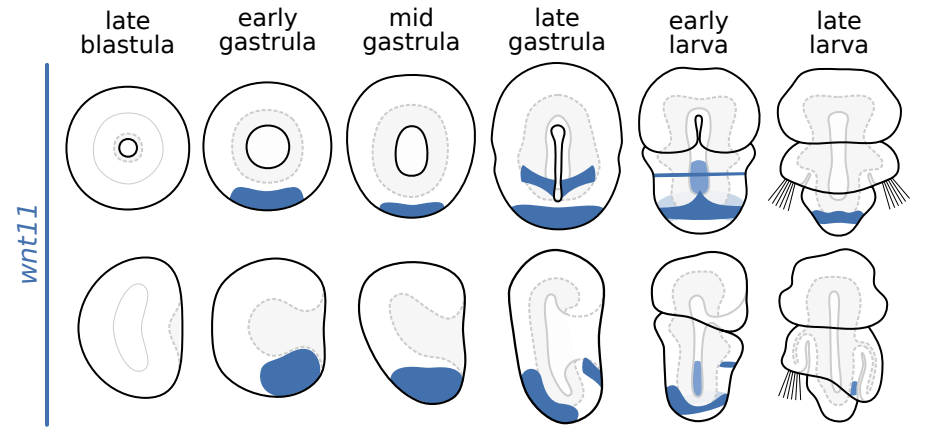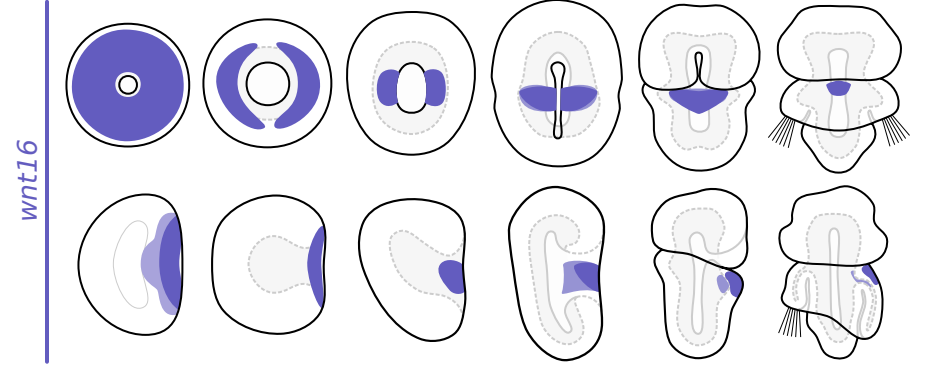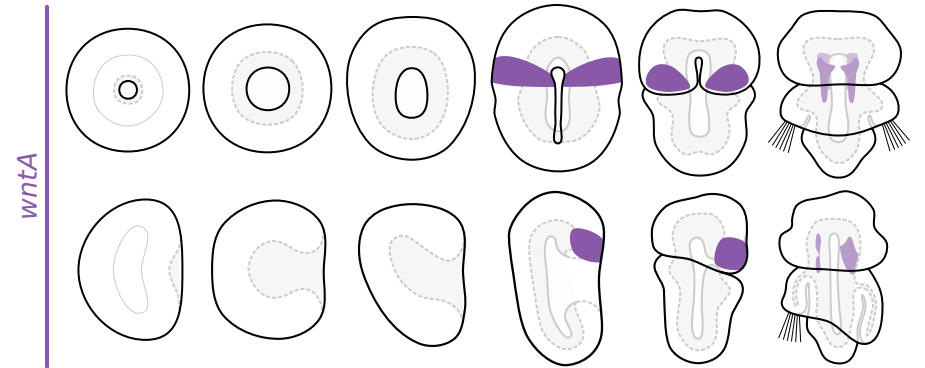

Supplement: Supplementary file 7 — Additional file 7: Fig. S7: [PDF] Schematic drawings summarizing the expression of wnt8, wnt9, wnt10, wnt11, wnt16, and wntA in Terebratalia transversa. For each developmental stage of each gene, a blastoporal/ventral view (top) and a lateral view (bottom) are shown. Faded colors represent expression domains in the mesoderm or endoderm, or in the ectoderm when it is beneath the mantle lobe. [file 12915_2024_1988_MOESM7_ESM.pdf]

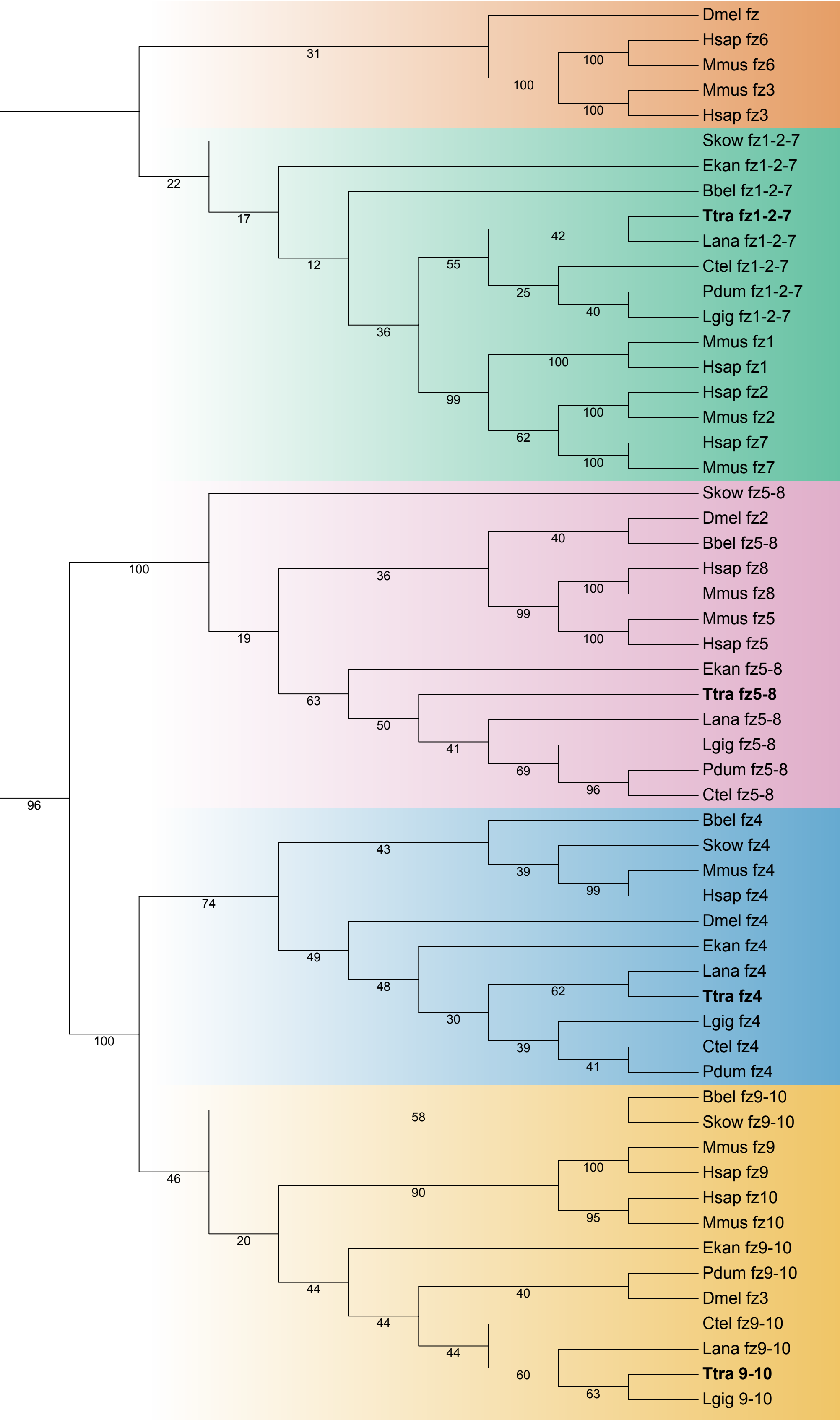

*fz3/6*

*fz1/2/7*

*fz5/8*

*fz4*

*fz9/10*

Supplement: Supplementary file 8 — Additional file 8: Fig. S8: [PDF] Orthology assignment of Terebratalia transversa Frizzled proteins. Best-scoring tree of a maximum likelihood phylogenetic analysis using the amino acid sequences of well-annotated Frizzled proteins. The color-coding represents different Frizzled subfamilies and the numbers show the support values of individual branches. Terebratalia transversa (Ttra) orthologs are highlighted in bold. The other species are Branchiostoma belcheri (Bbel), Capitella teleta (Ctel), Drosophila melanogaster (Dmel), Euperipatoides kanangrensis (Ekan), Homo sapiens (Hsap), Lingula anatina (Lana), Lottia gigantea (Lgig), Mus musculus (Mmus), Platynereis dumerilii (Pdum), and Saccoglossus kowalevskii (Skow). [file 12915_2024_1988_MOESM8_ESM.pdf]

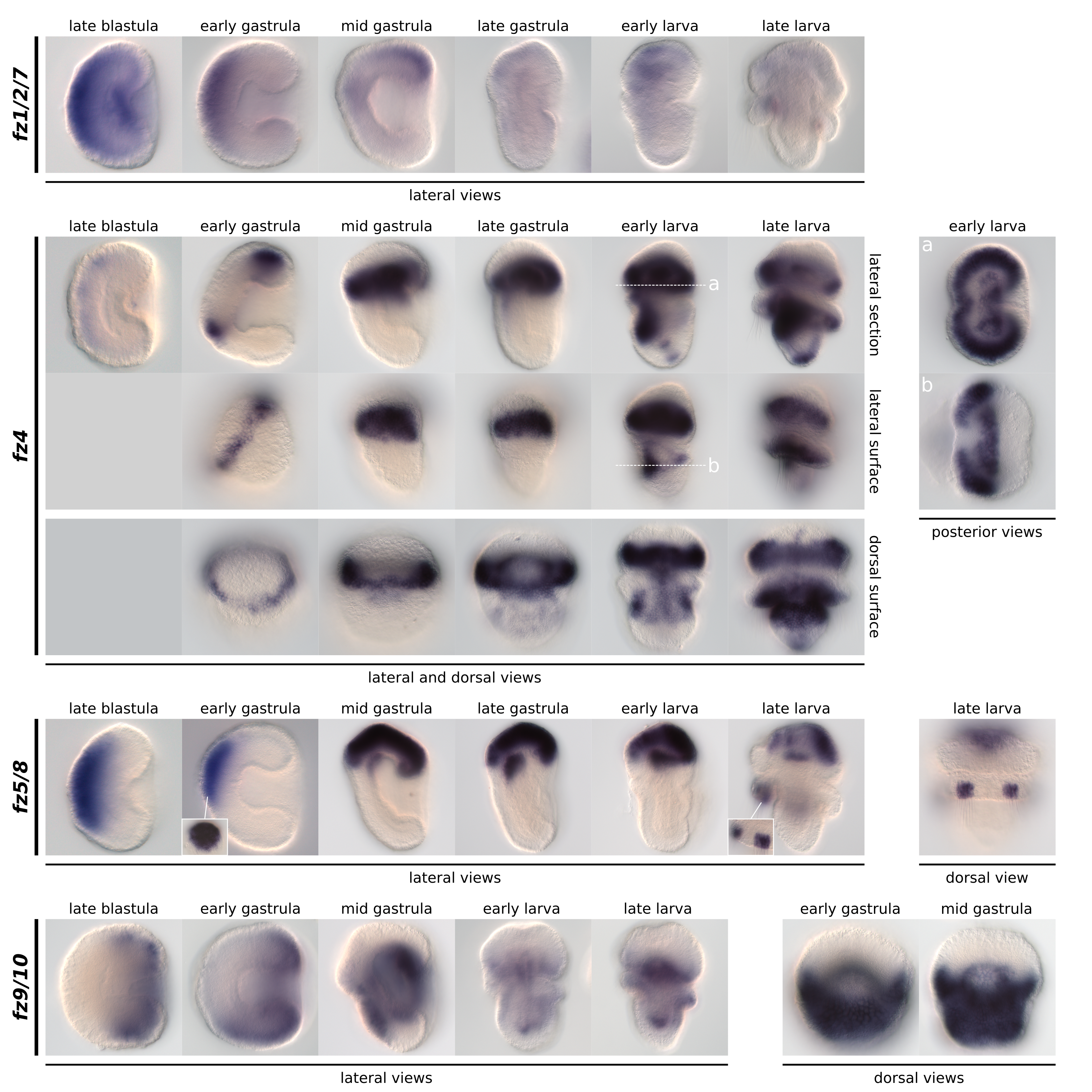

Supplement: Supplementary file 9 — Additional file 9: Fig. S9: [PNG] Whole-mount colorimetric in situ hybridization of Terebratalia transversa Frizzled genes. Additional views of fz1/2/7, fz4, fz5/8, and fz9/10 expression between late blastula and late larva. The panels show representative expression patterns for each sample. The stainings for fz1/2/7 in the samples from early gastrula to late larva are underdeveloped. Dashed lines indicate the position of the optical section shown in adjacent panels. [file 12915_2024_1988_MOESM9_ESM.png]

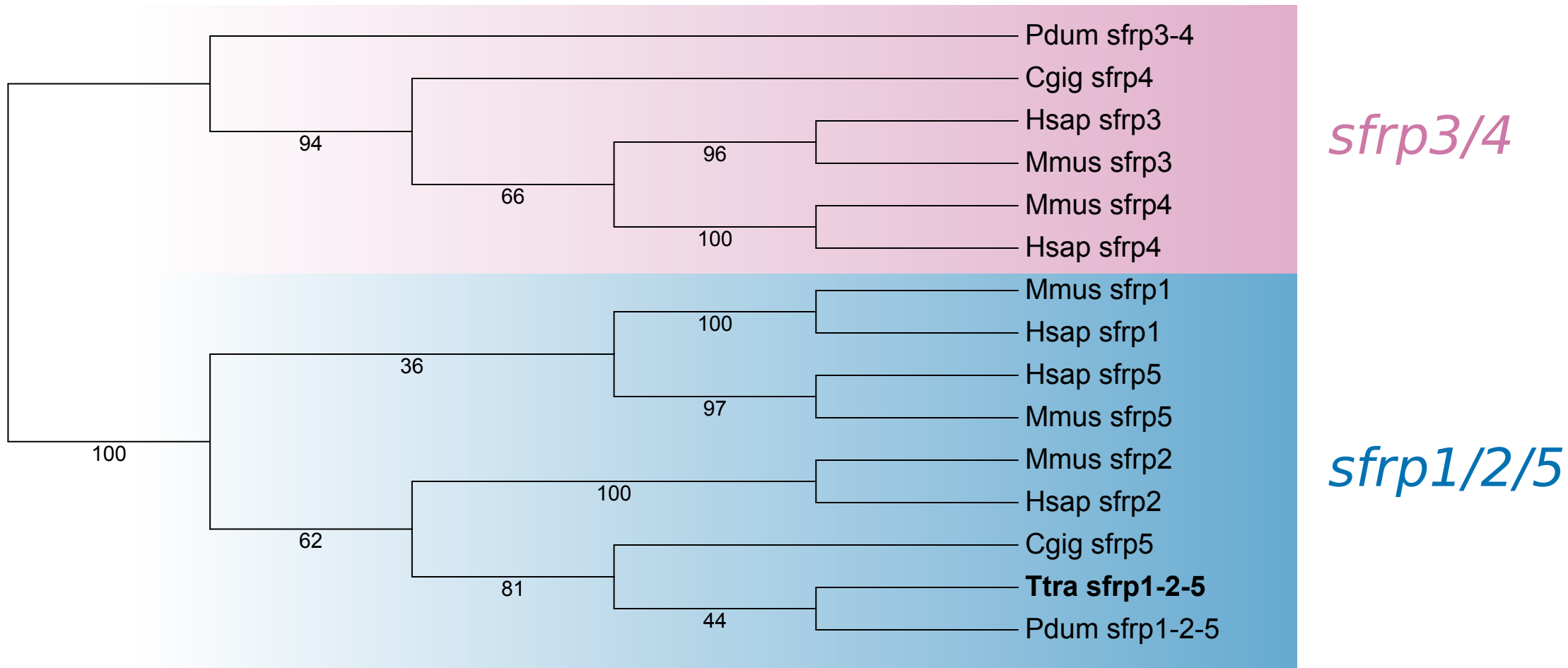

Supplement: Supplementary file 10 — Additional file 10: Fig. S10: [PDF] Orthology assignment of Terebratalia transversa sFRP proteins. Best-scoring tree of a maximum likelihood phylogenetic analysis using the amino acid sequences of sFRP genes. The color-coding represents different sFRP subfamilies and the numbers show the support values of individual branches. Terebratalia transversa (Ttra) ortholog is highlighted in bold. The other species are Homo sapiens (Hsap), Crassostrea gigantea (Cgig), Mus musculus (Mmus), and Platynereis dumerilii (Pdum). [file 12915_2024_1988_MOESM10_ESM.pdf]

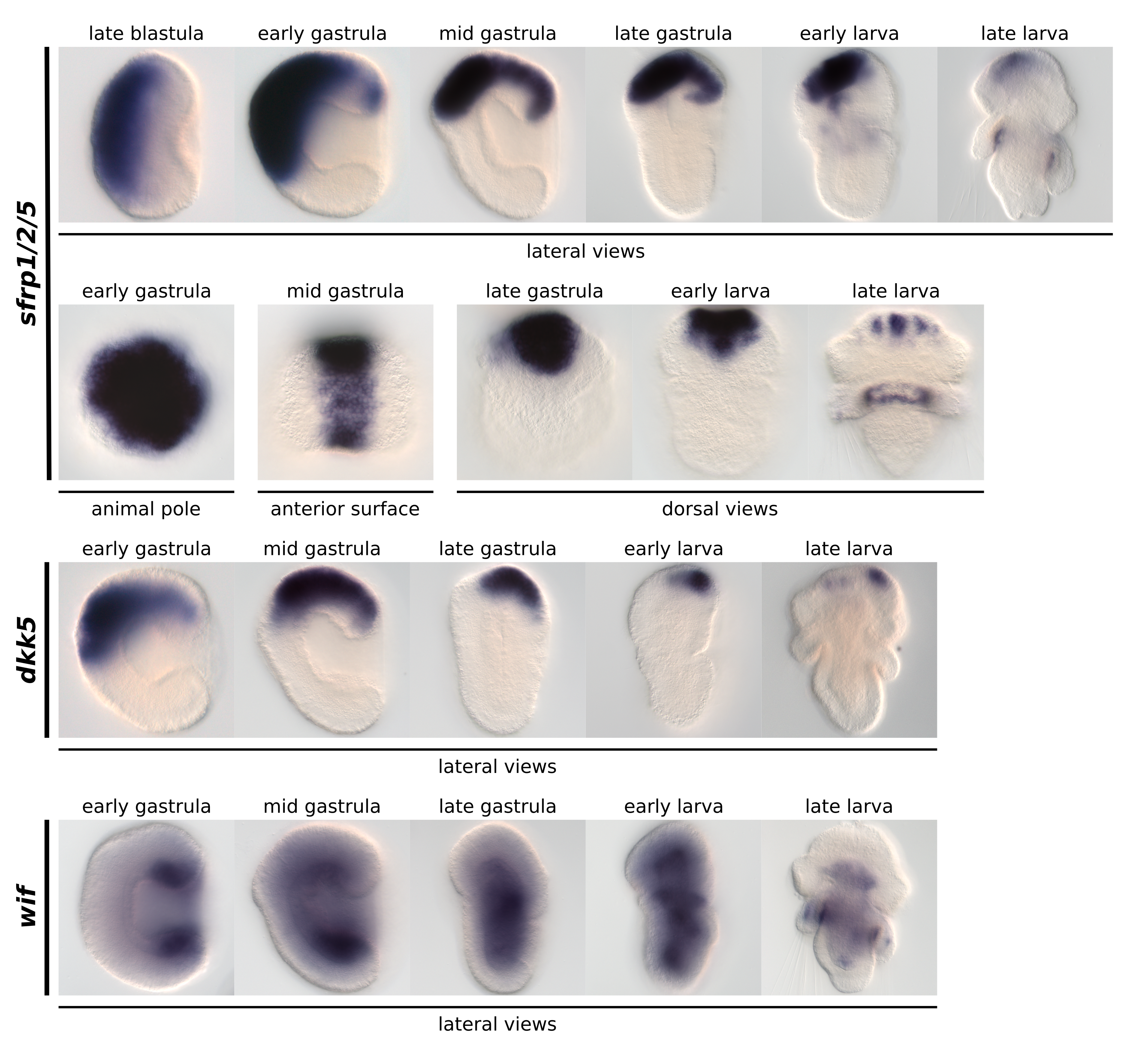

Supplement: Supplementary file 11 — Additional file 11: Fig. S11: [PNG] Whole-mount colorimetric in situ hybridization of Terebratalia transversa Wnt antagonists. Additional views of Wnt antagonists expression between late blastula and late larva. The panels show representative expression patterns for each sample. [file 12915_2024_1988_MOESM11_ESM.png]

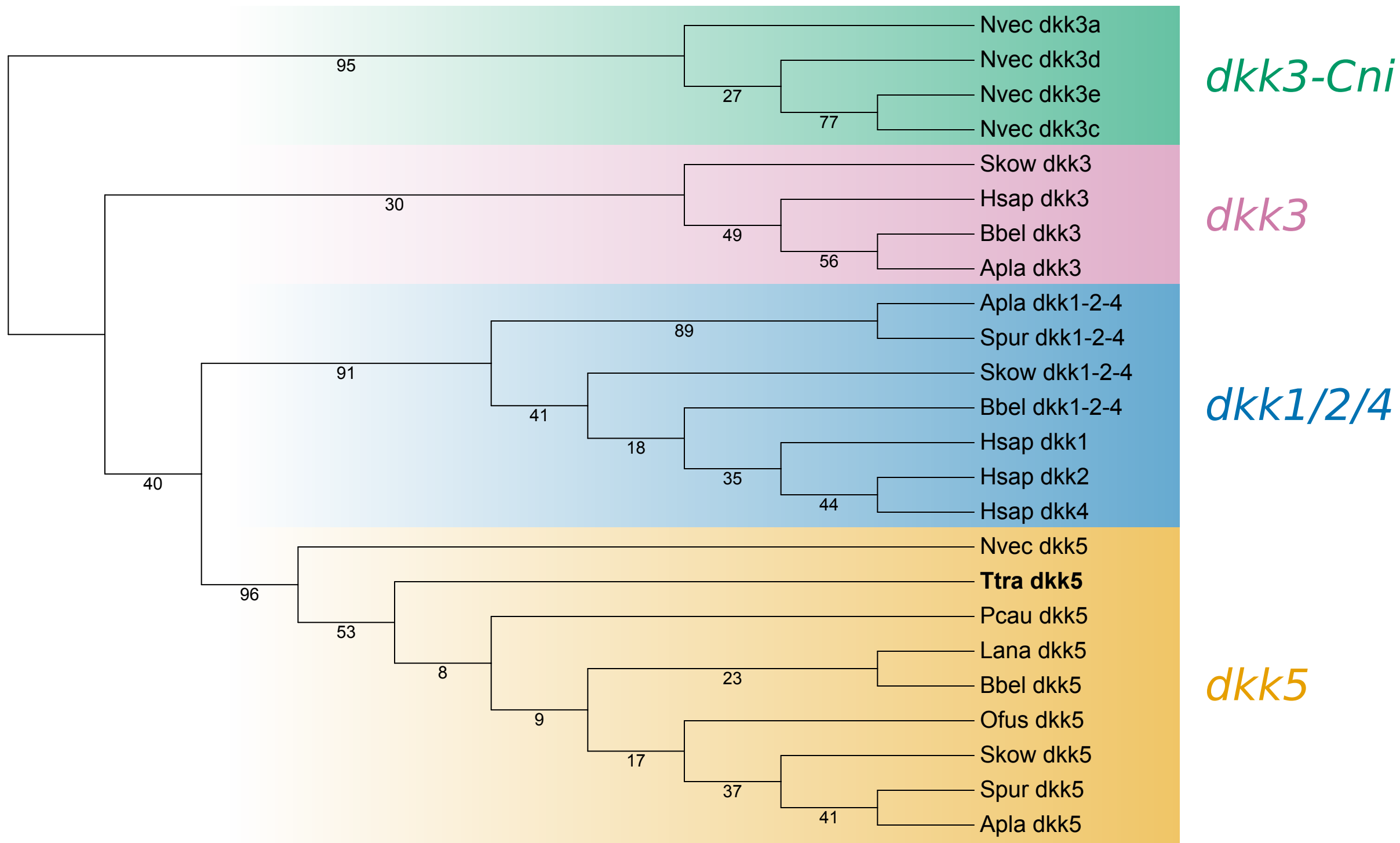

Supplement: Supplementary file 12 — Additional file 12: Fig. S12: [PDF] Orthology assignment of Terebratalia transversa Dkk proteins. Best-scoring tree of a maximum likelihood phylogenetic analysis using the amino acid sequences of Dkk from diverse metazoans. Color-coding represents different Dkk subfamilies. Numbers show support values of individual branches. Terebratalia transversa (Ttra) ortholog, highlighted in bold, groups with a previously unidentified Dkk subfamily, in addition to dkk3 and dkk1/2/4, which we named dkk5. Non-vertebrate deuterostomes such as the hemichordate Saccoglossus kowalevskii (Skow), the echinoderm Acanthaster planci (Apla), and the cephalochordate Branchiostoma belcheri (Bbel), have an ortholog of each Dkk family. Vertebrates lost dkk5. Protostomes lost dkk1/2/4 and dkk3 early on, but retained dkk5 in some lineages such as T. transversa, Priapulus caudatus (Pcau), and Owenia fusiformis (Ofus). Cnidarians expanded dkk3 but lost dkk1/2/4. Overall, this suggests dkk1/2/4, dkk3, and dkk5 were the ancestral subfamilies in the Cnidaria–bilaterian branch. The other species are Homo sapiens (Hsap), Lingula anatina (Lana), Nematostella vectensis (Nvec), and Strongylocentrotus purpuratus (Spur). [file 12915_2024_1988_MOESM12_ESM.pdf]

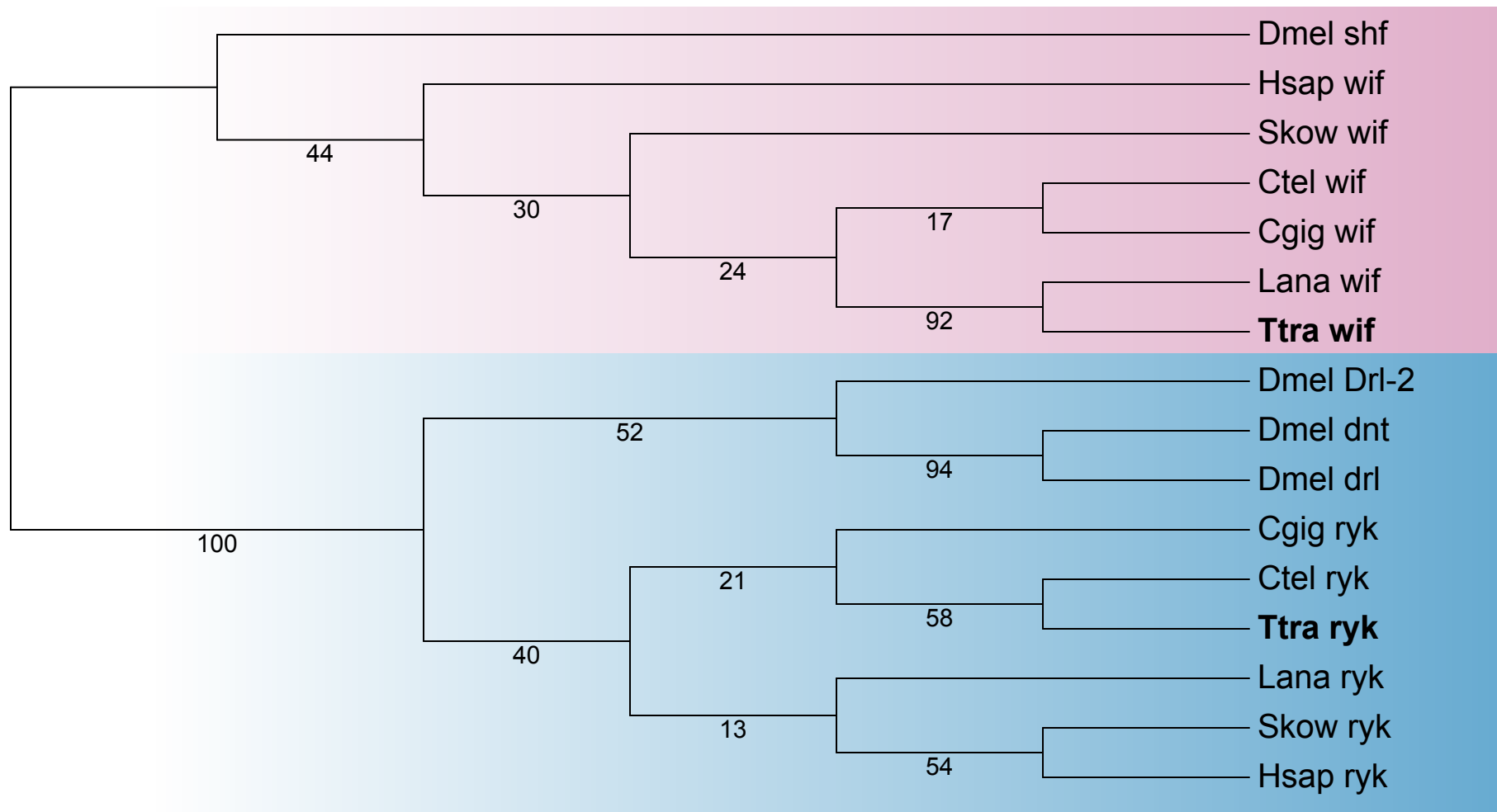

*wif*

*ryk*

Supplement: Supplementary file 13 — Additional file 13: Fig. S13: [PDF] Orthology assignment of Terebratalia transversa Wif proteins. Best-scoring tree of a maximum likelihood phylogenetic analysis using the amino acid sequences of known Wif proteins (Wnt inhibitory factor). As an outgroup, we used the tyrosine-protein kinase Ryk which also has a WIF domain. The color-coding represents Wif and Ryk families. Numbers show the support values of individual branches. Terebratalia transversa (Ttra) orthologs are highlighted in bold. The other species are Capitella teleta (Ctel), Crassostrea gigantea (Cgig), Drosophila melanogaster (Dmel), Homo sapiens (Hsap), Lingula anatina (Lana), and Saccoglossus kowalevskii (Skow). [file 12915_2024_1988_MOESM13_ESM.pdf]

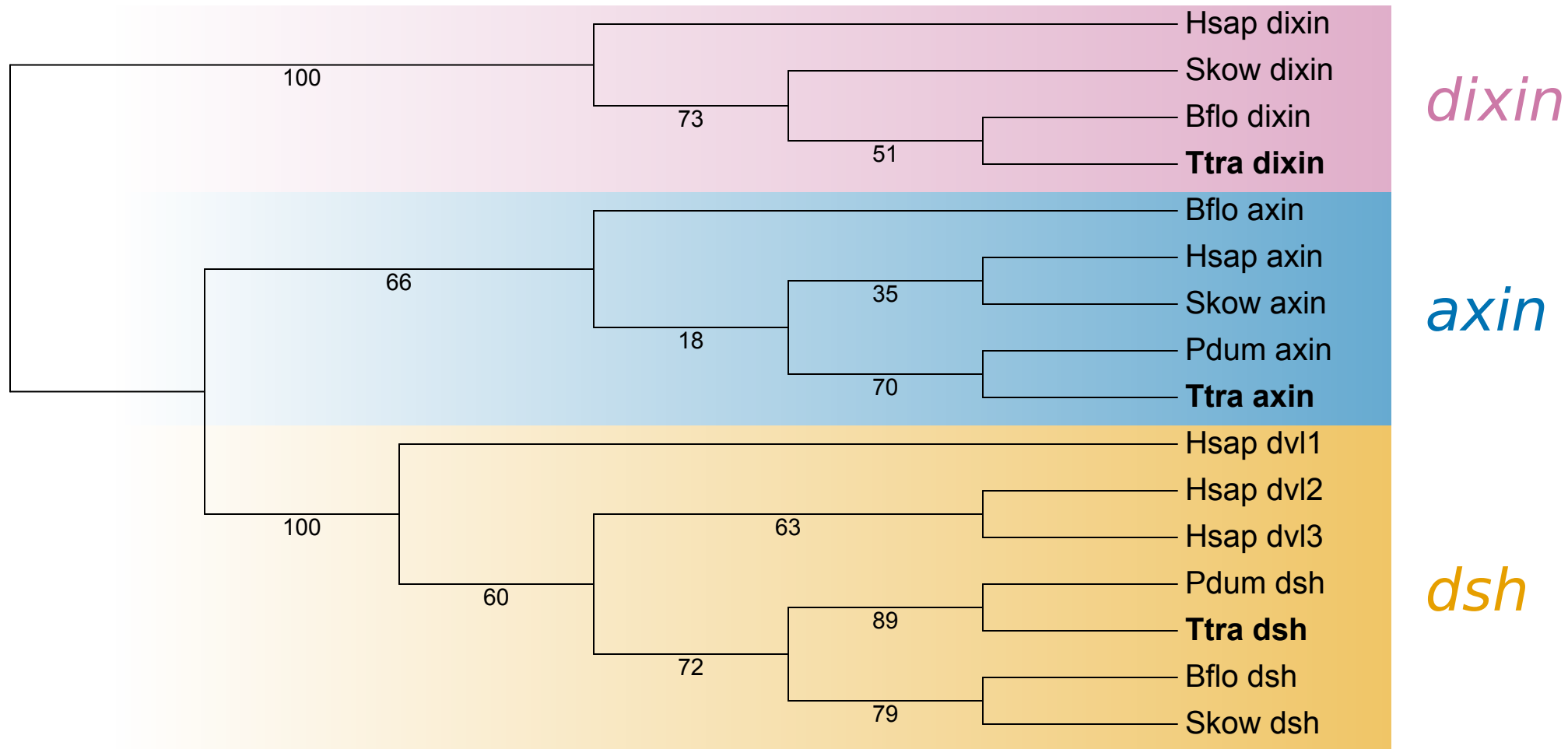

Supplement: Supplementary file 14 — Additional file 14: Fig. S14: [PDF] Orthology assignment of Terebratalia transversa Dsh proteins. Best-scoring tree of a maximum likelihood phylogenetic analysis using the amino acid sequences of known Dsh, Axin, and Dixin proteins. The three belong to the DIX domain superfamily. Each family is color-coded, and the numbers show support values of individual branches. Terebratalia transversa (Ttra) orthologs are highlighted in bold. The other species are Branchiostoma floridae (Bflo), Homo sapiens (Hsap), Platynereis dumerilii (Pdum), and Saccoglossus kowalevskii (Skow). [file 12915_2024_1988_MOESM14_ESM.pdf]

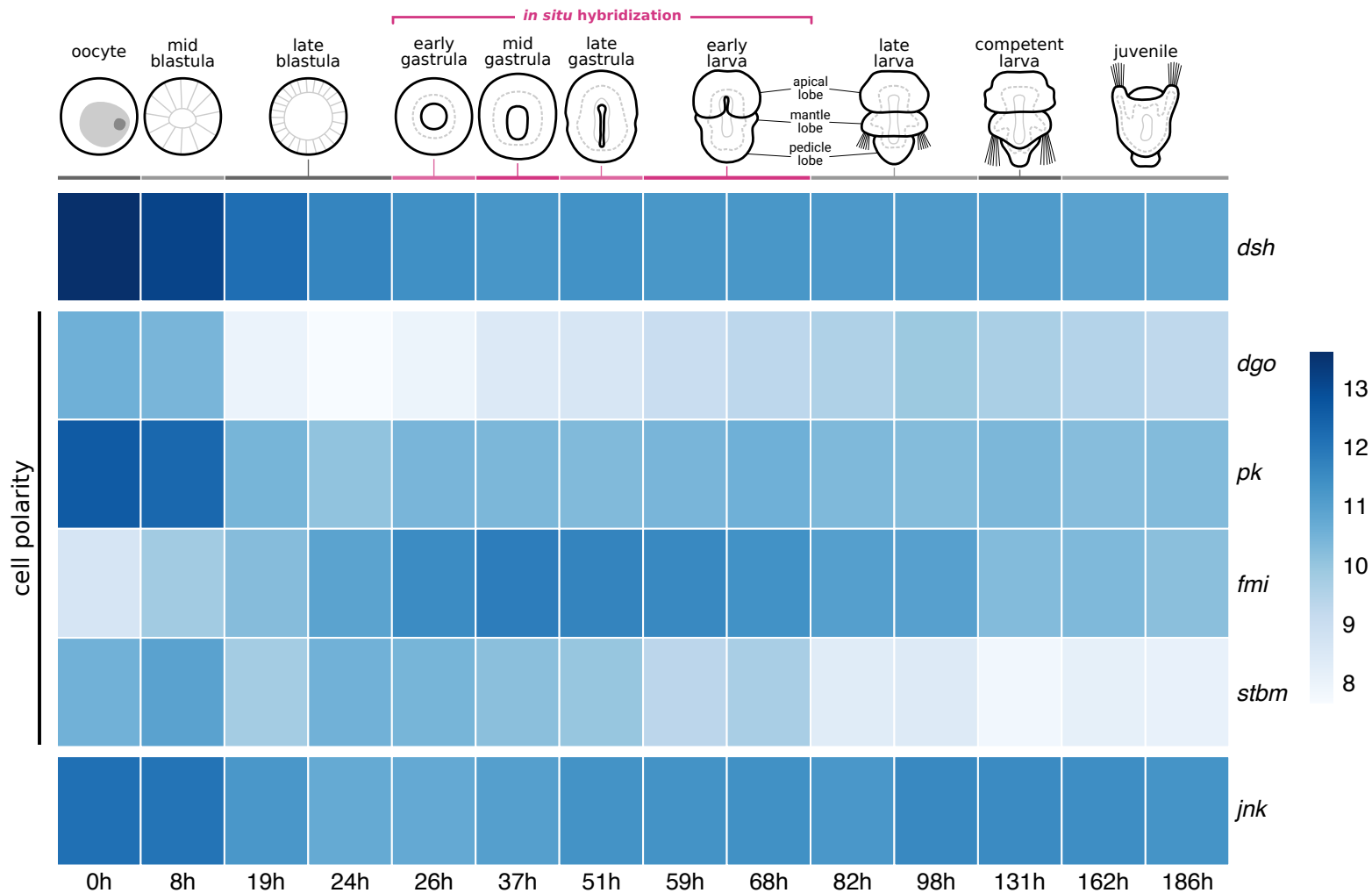

Supplement: Supplementary file 15 — Additional file 15: Fig. S15: [PDF] Expression of Wnt/PCP pathway during Terebratalia transversa development. The heatmap represents the log-normalized transcript counts for dsh, dgo, pk, fmi, stbm, and jnk calculated from stage-specific RNA-Seq data. Each cell shows the average value between two replicates. The illustrations depict T. transversa developmental stages from the oocyte until the post-metamorphic juvenile. The stages we analyzed using in situ hybridization (early gastrula to late larva) are highlighted in magenta. [file 12915_2024_1988_MOESM15_ESM.pdf]

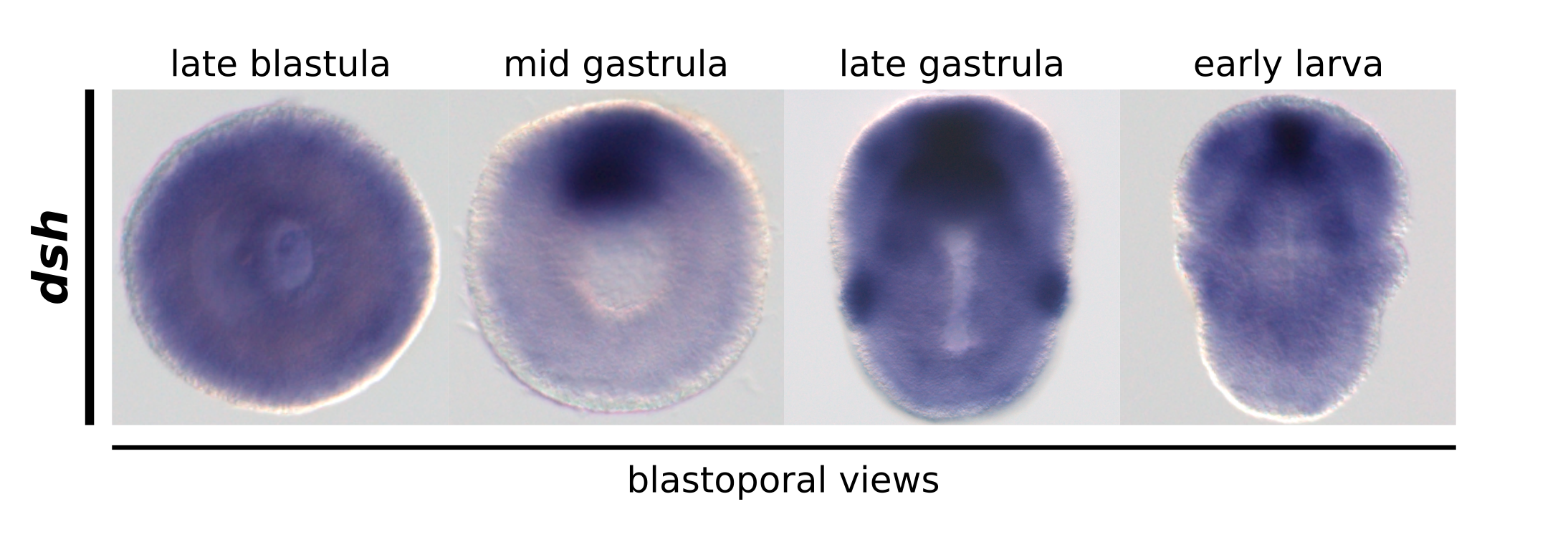

Supplement: Supplementary file 16 — Additional file 16: Fig. S16: [PNG] Over-developed whole-mount colorimetric in situ hybridization of Terebratalia transversa dsh gene. The longer reaction time reveals that dsh transcripts are ubiquitously expressed in most embryonic tissues. [file 12915_2024_1988_MOESM16_ESM.png]

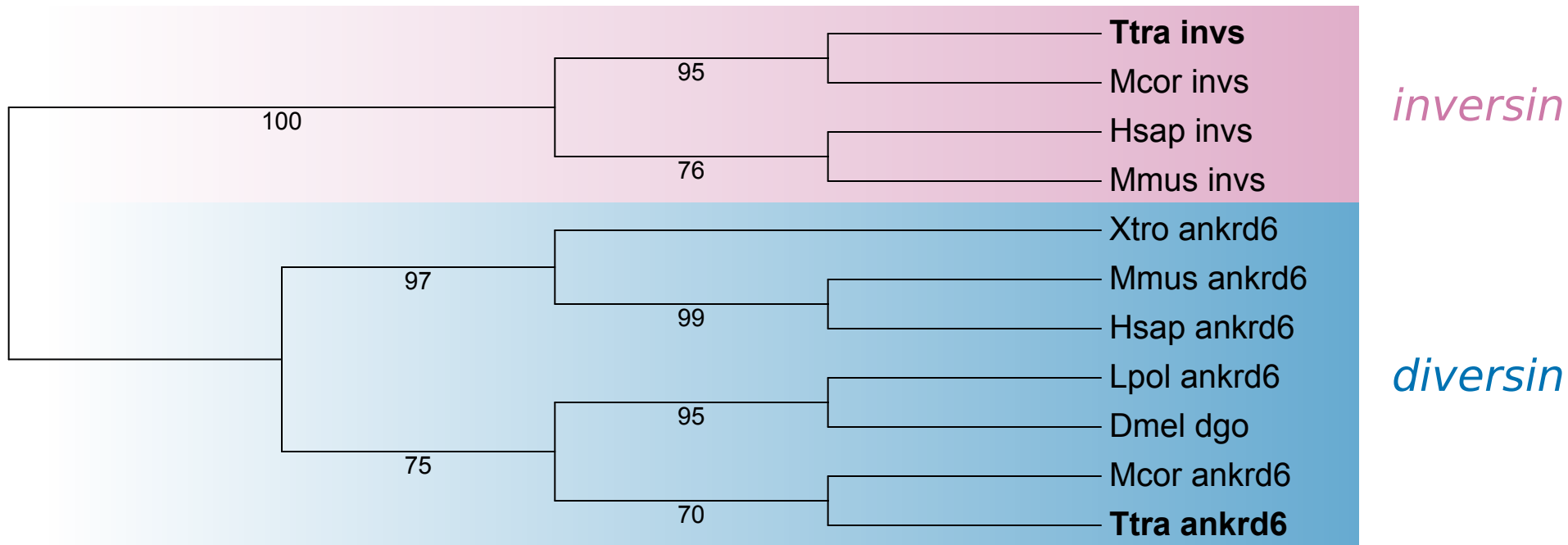

Supplement: Supplementary file 17 — Additional file 17: Fig. S17: [PDF] Orthology assignment of Terebratalia transversa Dgo proteins. Best-scoring tree of a maximum likelihood phylogenetic analysis using the amino acid sequences of Dgo (ANKRD6 or Diversin). We used Inversin proteins as an outgroup since they also have ankyrin repeats. Terebratalia transversa (Ttra) orthologs are highlighted in bold. The other species are Drosophila melanogaster (Dmel), Homo sapiens (Hsap), Limulus polyphemus (Lpol), Mus musculus (Mmus), Mytilus coruscus (Mcor), and Xenopus tropicalis (Xtro). [file 12915_2024_1988_MOESM17_ESM.pdf]

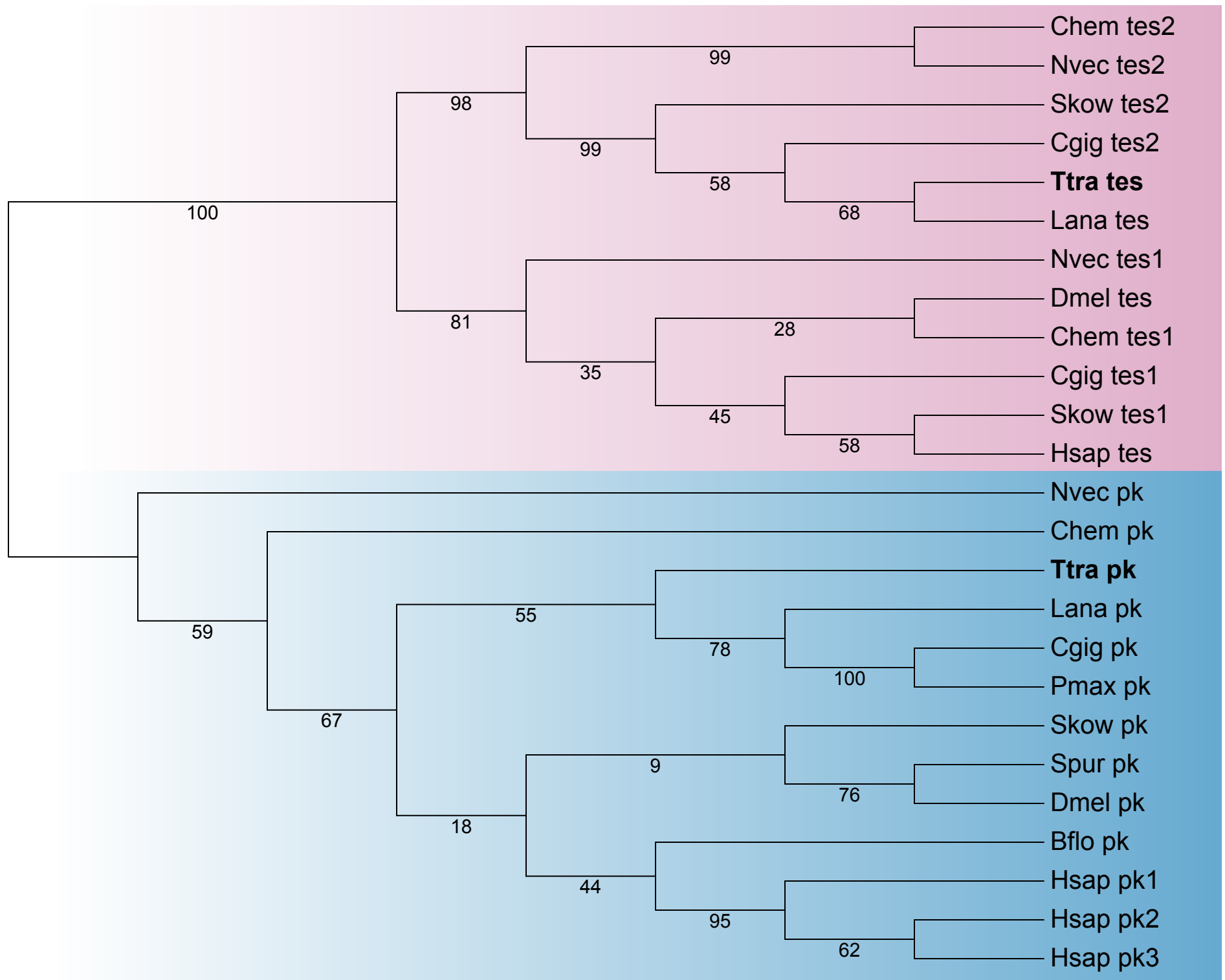

*testin*

*prickle*

Supplement: Supplementary file 18 — Additional file 18: Fig. S18: [PDF] Orthology assignment of Terebratalia transversa Pk proteins. Best-scoring tree of a maximum likelihood phylogenetic analysis using the amino acid sequences of Pk from diverse metazoans. As an outgroup, we used Testin, a related protein which also contains a LIM and a PET domain.Terebratalia transversa (Ttra) orthologs are highlighted in bold. The other species are Clytia hemisphaerica (Chem), Crassostrea gigantea (Cgig), Drosophila melanogaster (Dmel), Homo sapiens (Hsap), Lingula anatina (Lana), Nematostella vectensis (Nvec), Pecten maximus (Pmax), and Saccoglossus kowalevskii (Skow). [file 12915_2024_1988_MOESM18_ESM.pdf]

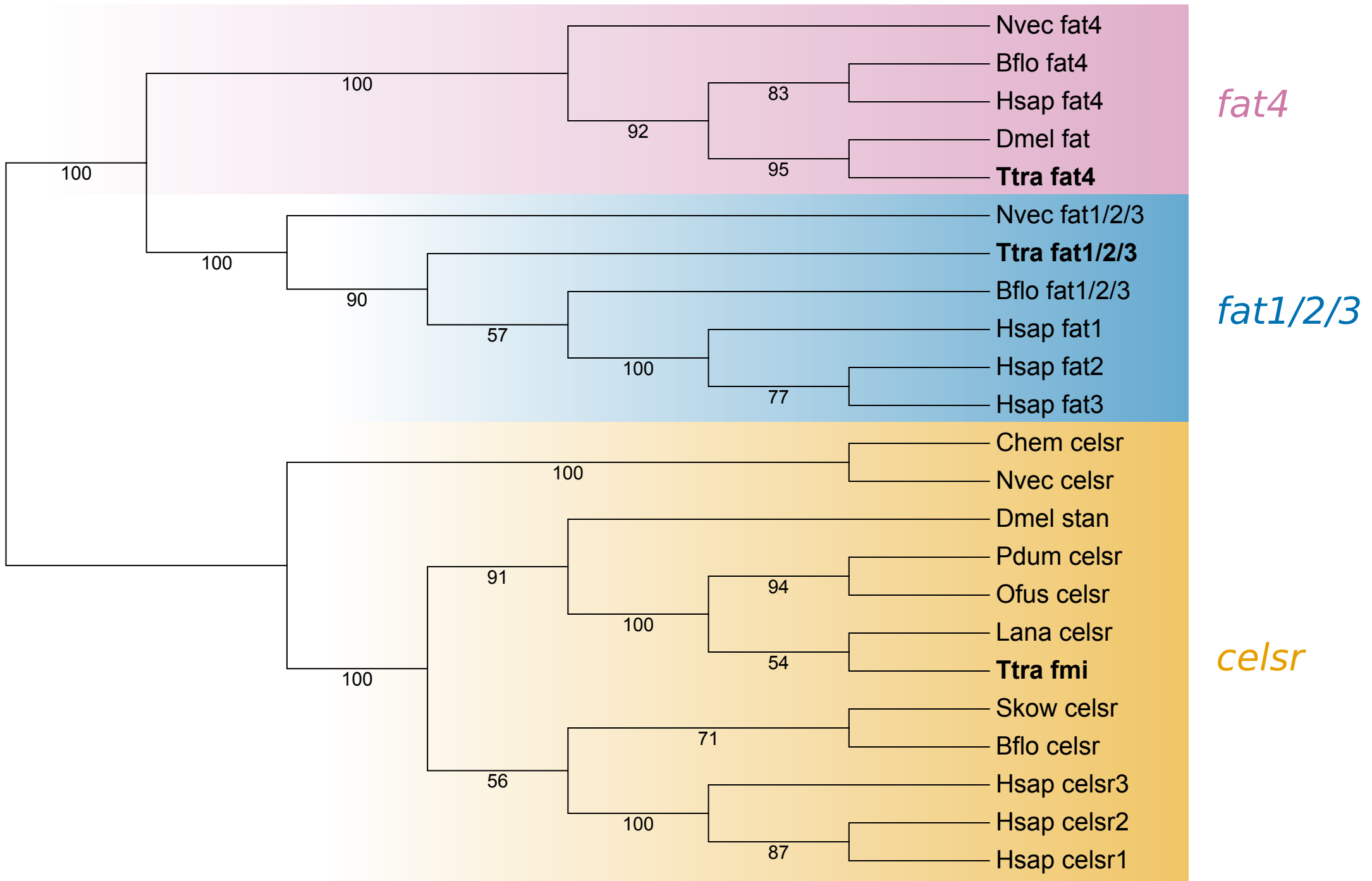

Supplement: Supplementary file 19 — Additional file 19: Fig. S19: [PDF] Orthology assignment of Terebratalia transversa Fmi proteins. Best-scoring tree of a maximum likelihood phylogenetic analysis using the amino acid sequences of Fmi. As outgroups, we used the related Fat family protocadherins which also contain cadherin and laminin domains. Terebratalia transversa (Ttra) orthologs are highlighted in bold. The other species are Branchiostoma floridae (Bflo), Clytia hemisphaerica (Chem), Drosophila melanogaster (Dmel), Homo sapiens (Hsap), Lingula anatina (Lana), Nematostella vectensis (Nvec), Owenia fusiformis (Ofus), Platynereis dumerilii (Pdum), and Saccoglossus kowalevskii (Skow). [file 12915_2024_1988_MOESM19_ESM.pdf]

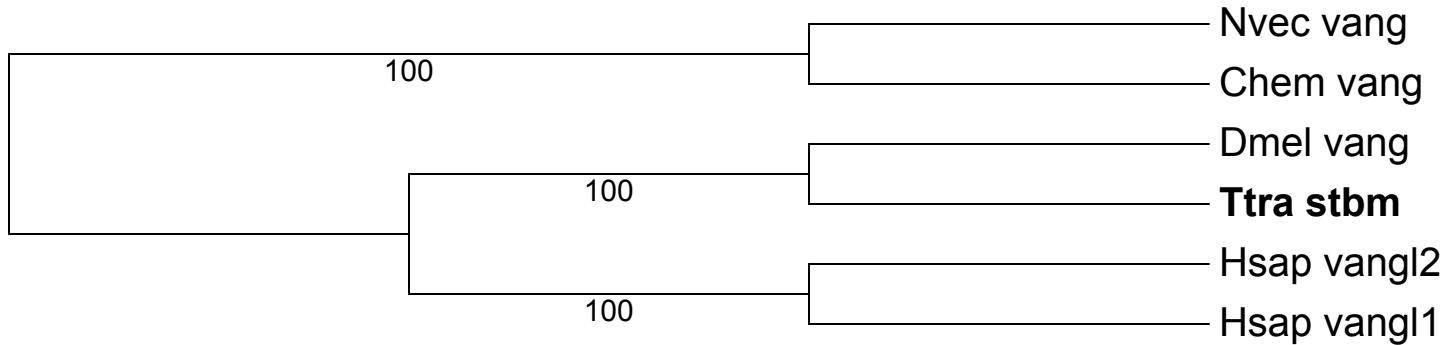

Supplement: Supplementary file 20 — Additional file 20: Fig. S20: [PDF] Orthology assignment of Terebratalia transversa Stbm proteins. Best-scoring tree of a maximum likelihood phylogenetic analysis using the amino acid sequences of Stbm from selected metazoans. Terebratalia transversa (Ttra) ortholog is highlighted in bold. The other species are Clytia hemisphaerica (Chem), Drosophila melanogaster (Dmel), Homo sapiens (Hsap), and Nematostella vectensis (Nvec). [file 12915_2024_1988_MOESM20_ESM.pdf]

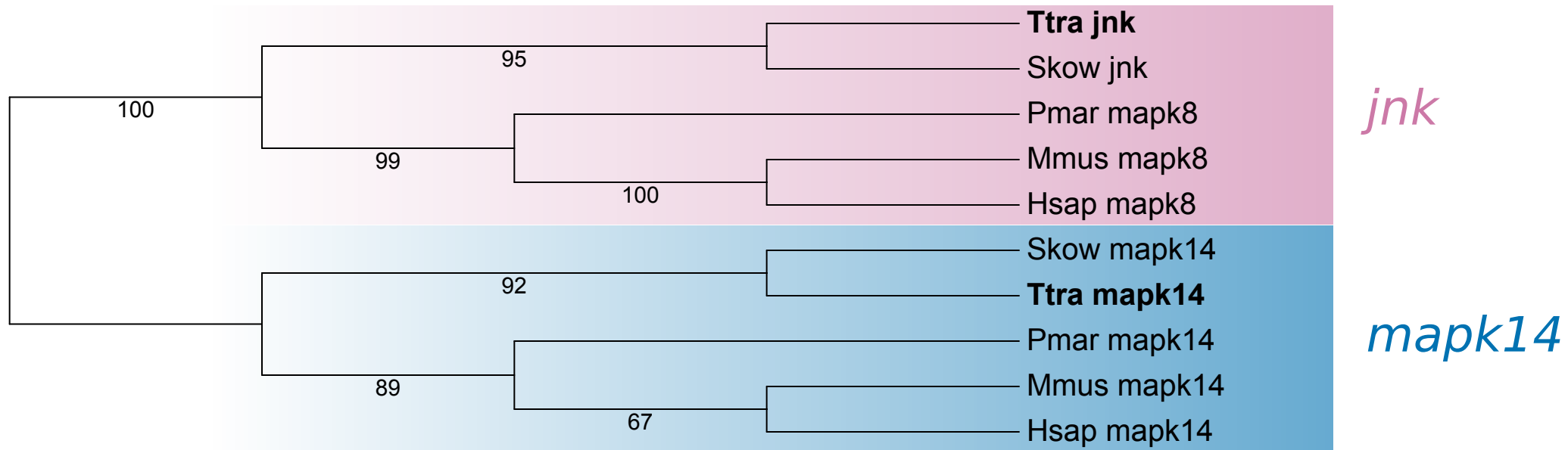

Supplement: Supplementary file 21 — Additional file 21: Fig. S21: [PDF] Orthology assignment of Terebratalia transversa Jnk proteins. Best-scoring tree of a maximum likelihood phylogenetic analysis using the amino acid sequences of Jnk. As outgroup, we used the related protein Mapk14. Terebratalia transversa (Ttra) orthologs are highlighted in bold. The other species are Homo sapiens (Hsap), Mus musculus (Mmus), Petromyzon marinus (Pmar), and Saccoglossus kowalevskii (Skow). [file 12915_2024_1988_MOESM21_ESM.pdf]

A

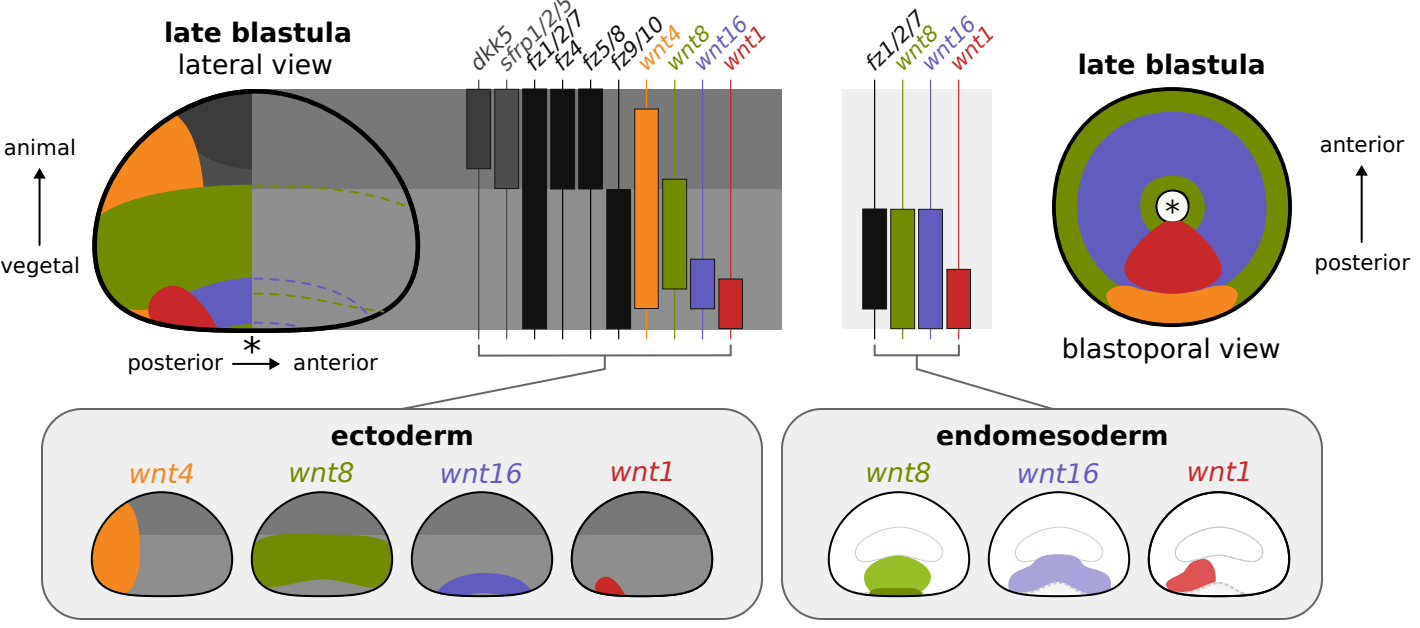

B

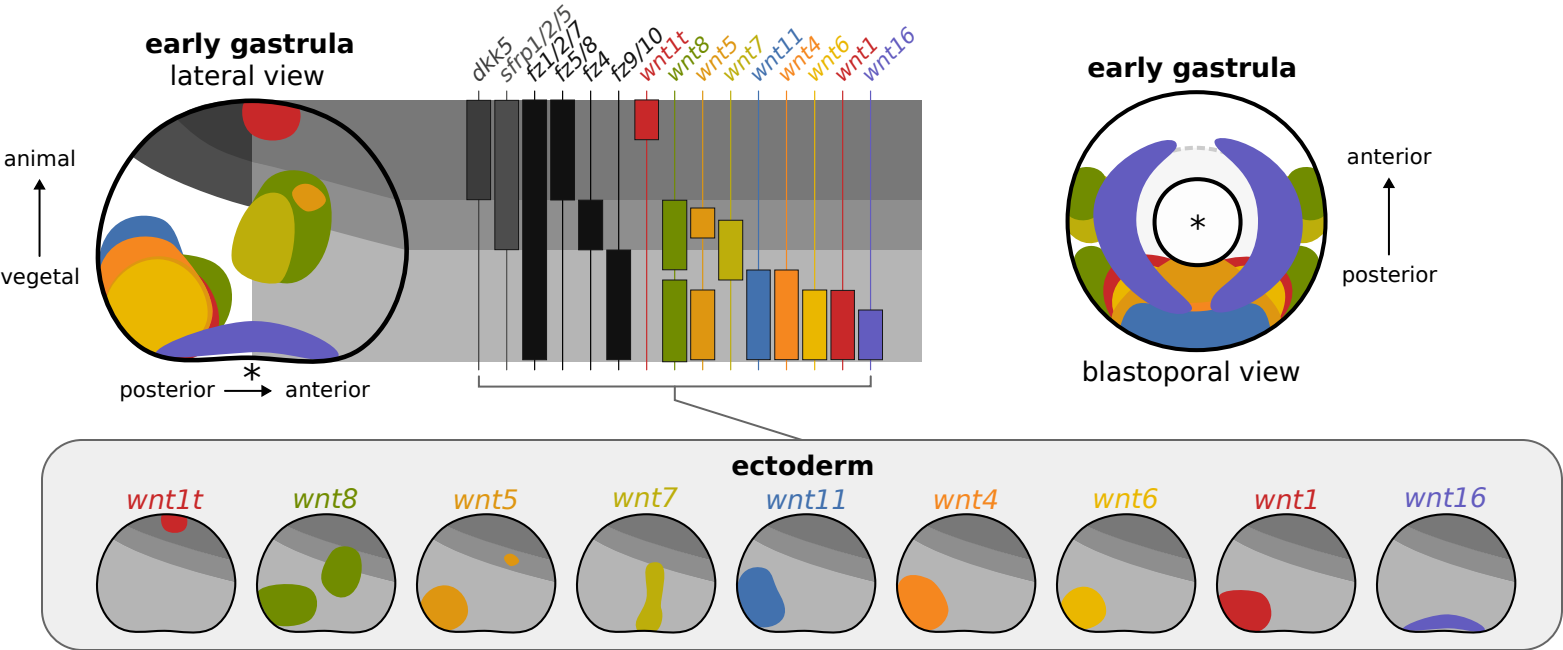

C

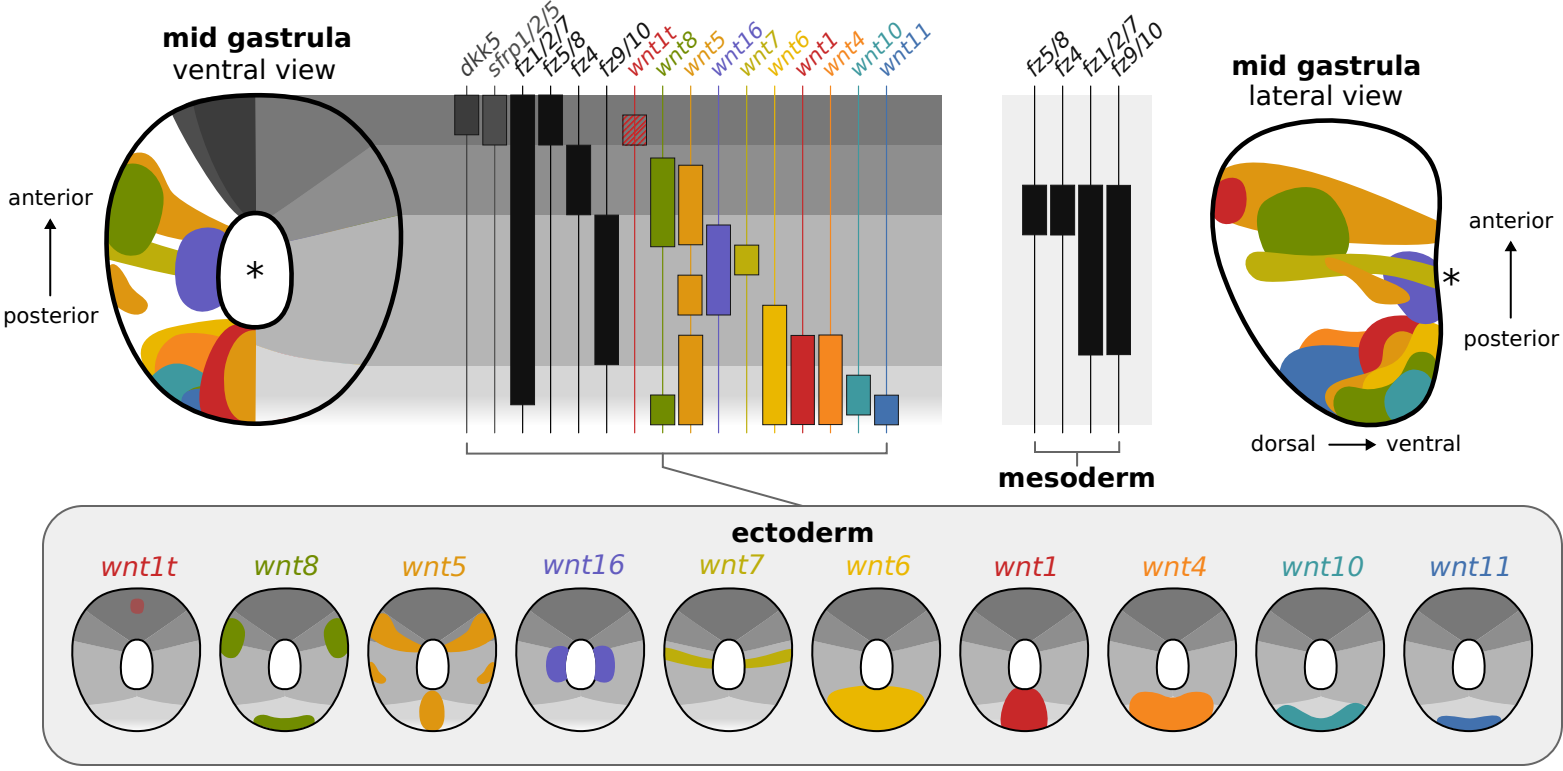

Supplement: Supplementary file 22 — Additional file 22: Fig. S22: [PDF] Summary of Terebratalia transversa Wnt signaling landscape during early gastrulation. Schematic drawings of Wnt genes colored by subfamilies, Frizzled genes by lighter shades of gray, and antagonists by darker shades of gray. The spatial localization of expression domains is superimposed on the embryo (left) and projected to highlight the individualized Wnt genes within the different transcriptional subregions grouped by germ layer (right). The gray boxes show the pattern of individual genes mapped to the embryo for clearer visualization of overlapping domains. (A) Late blastula in lateral and blastoporal views. (B) Early gastrula in lateral and blastoporal views. (C) Mid gastrula in blastoporal/ventral and lateral views. [file 12915_2024_1988_MOESM22_ESM.pdf]

A

## *Terebratalia*

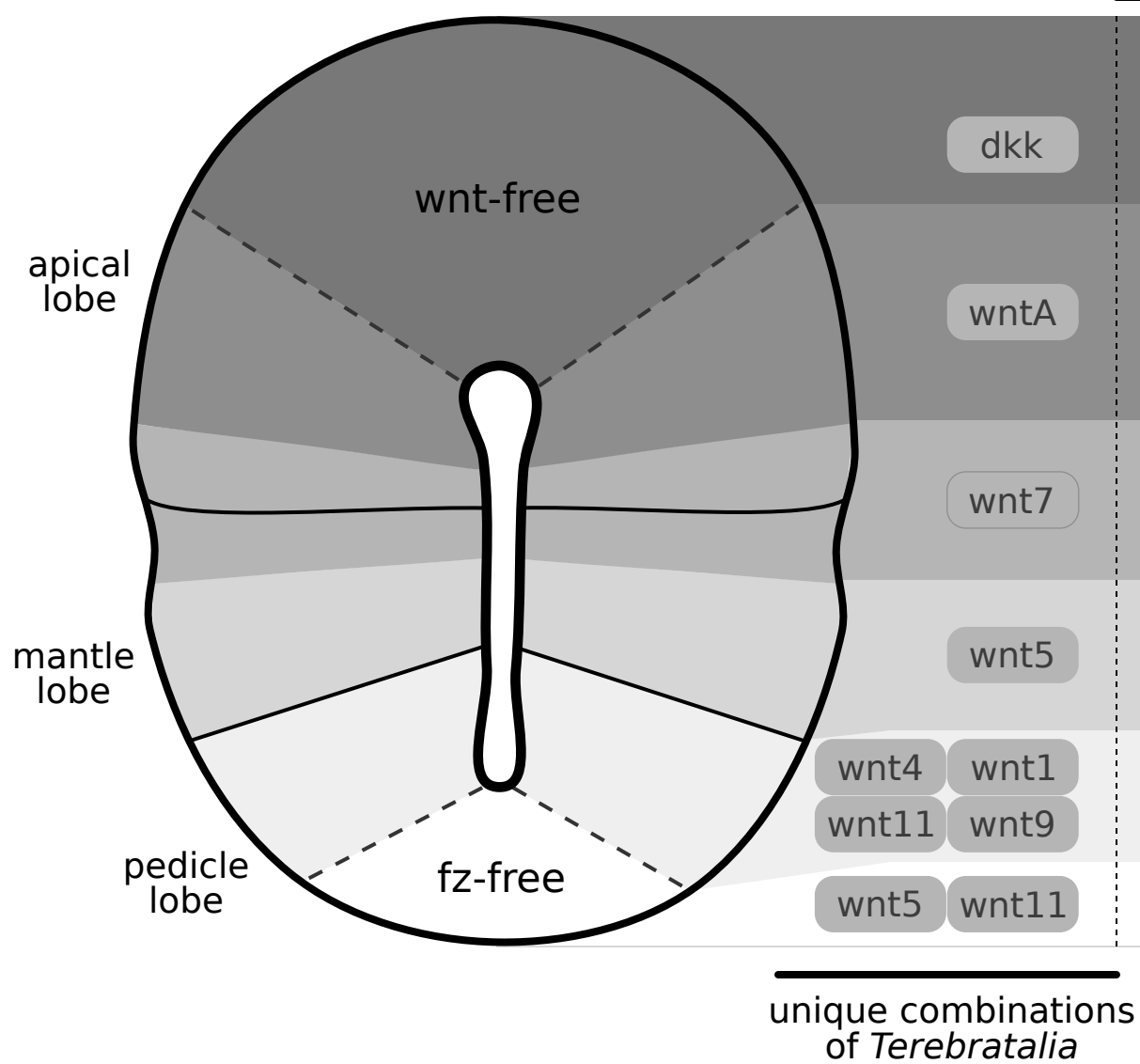

shared ligand-receptor combinations

## *Saccoglossus*

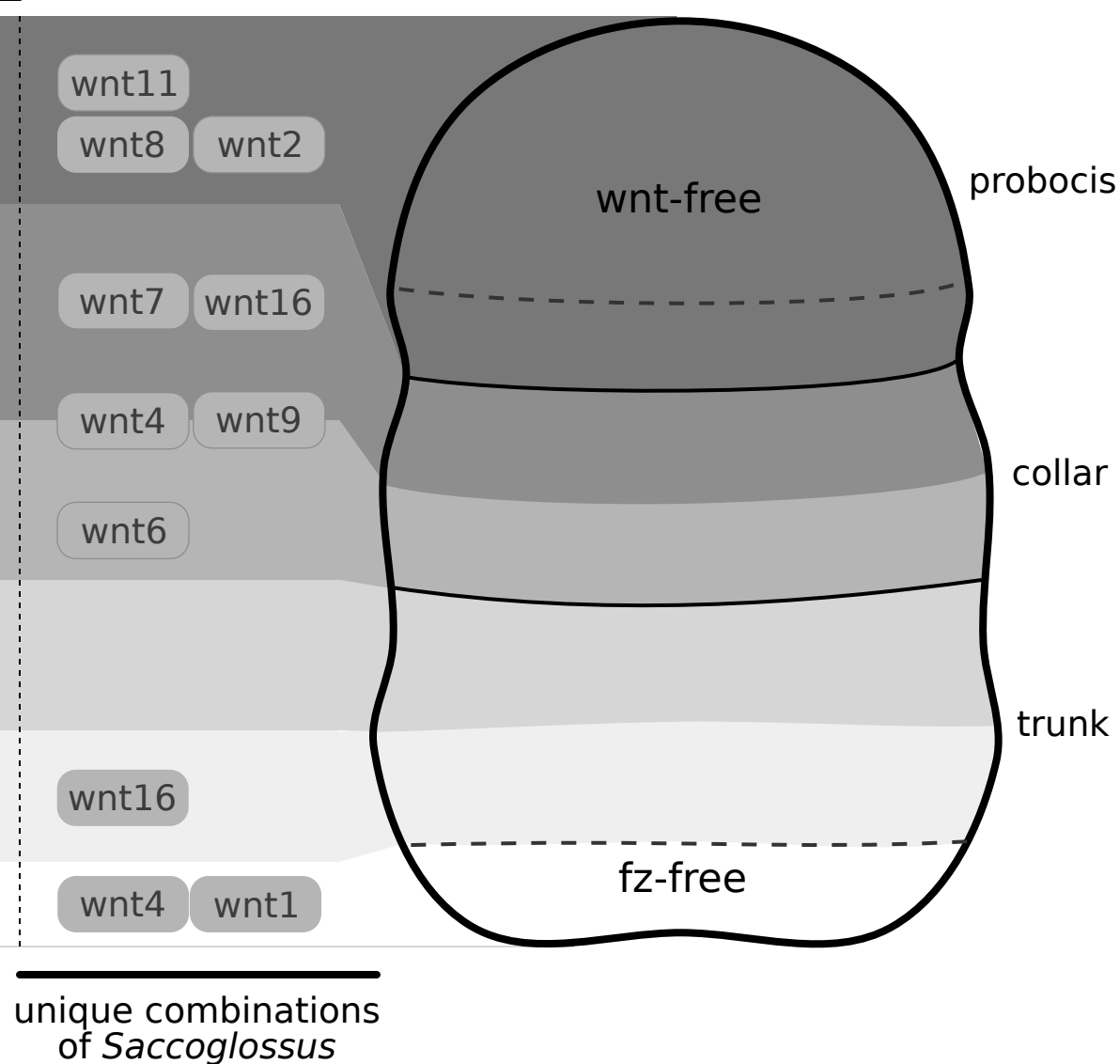

B

## Generalized ancestor

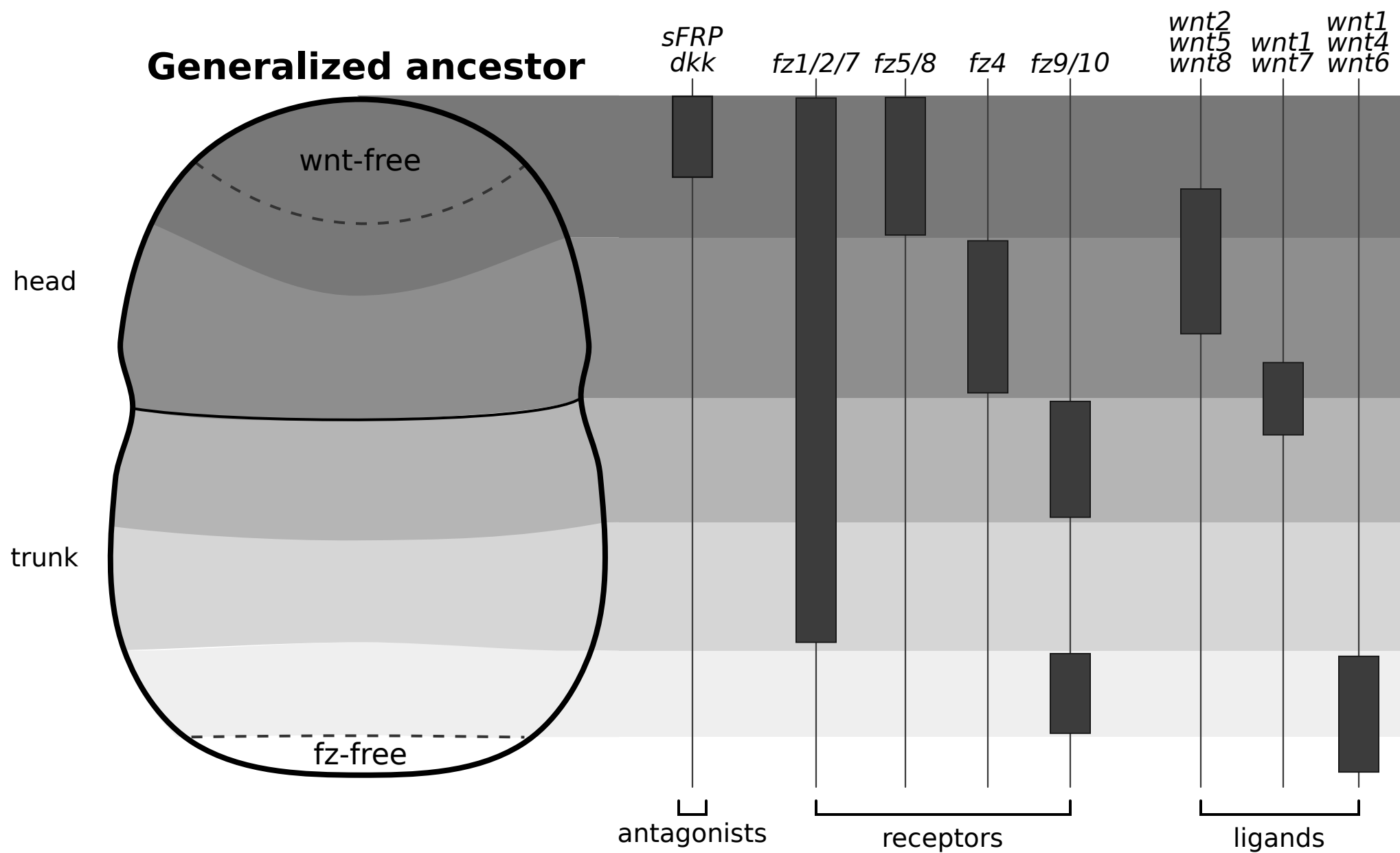

Supplement: Supplementary file 23 — Additional file 23: Fig. S23: [PDF] Wnt signaling ligand–receptor contexts compared between Terebratalia transversa and Saccoglossus kowalevskii. (A) Detailed comparison of shared and unique combinations of Wnt signaling components in brachiopod and hemichordate embryos. Solid lines represent morphological boundaries for the apical, mantle, and pedicle lobes, and dashed lines represent boundaries between transcriptional subregions. (B) Generalized ancestor showing the conserved Wnt subregions along the anteroposterior axis of T. transversa and S. kowalevskii. [file 12915_2024_1988_MOESM23_ESM.pdf]

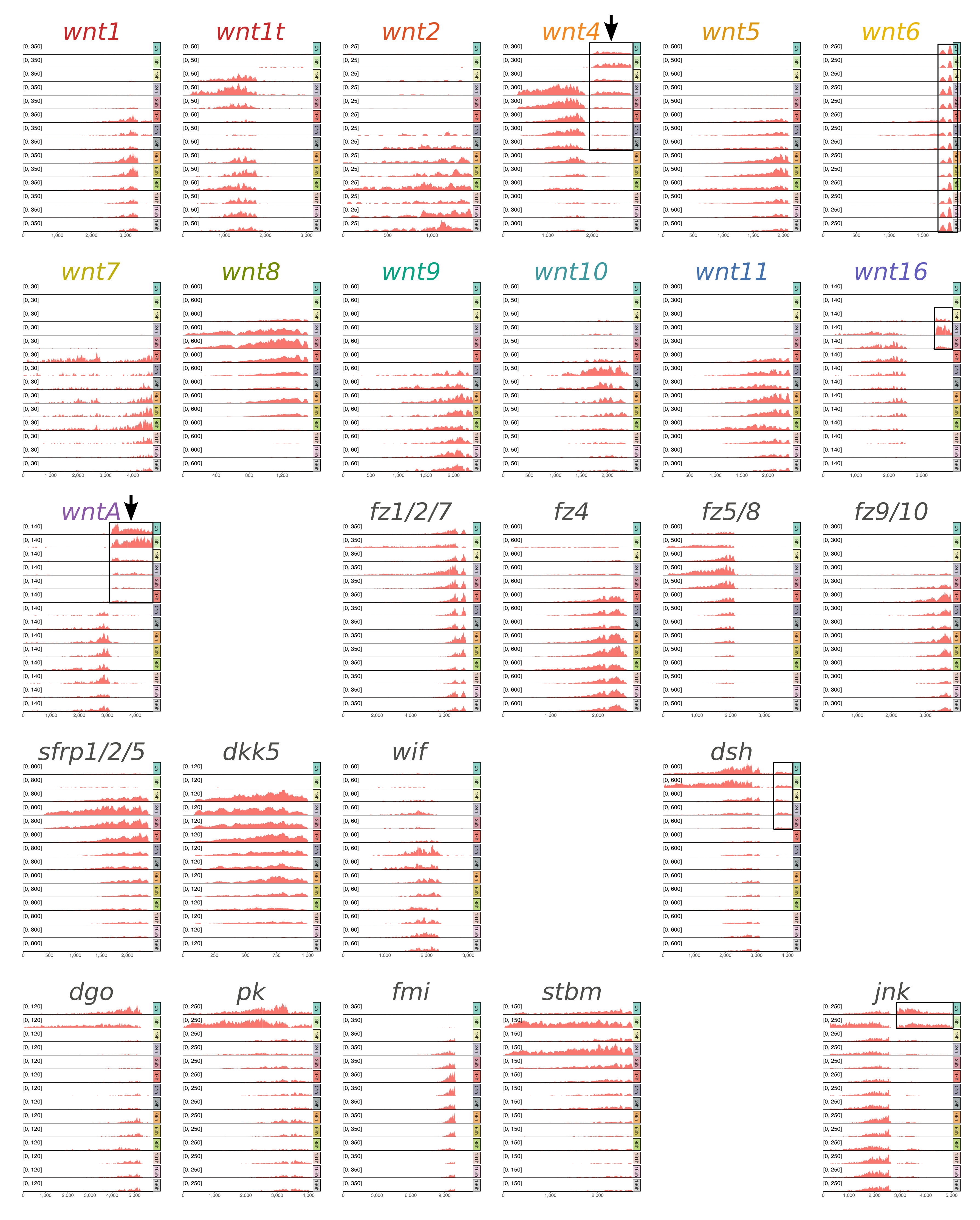

Supplement: Supplementary file 24 — Additional file 24: Fig. S24: [PNG] Read coverage of the stage-specific transcriptome mapped to the transcripts of Terebratalia transversa Wnt signaling components. Each gene shows the read coverage of one replicate along the transcript length for the 14 developmental stages sampled in this study (0–186 h). The Y axes are fixed to the maximum observed coverage of a gene (which is different for each gene). The black boxes highlight regions of uneven coverage. Arrows indicate the two cases, wnt4 and wntA, where the uneven coverage caused a bias in the quantification of expression levels. Although wnt6, wnt16, dsh, and jnk also show regions of uneven coverage, these reads did not alter the main expression profile of the gene. See the Methods section for more details. [file 12915_2024_1988_MOESM24_ESM.png]
