# Supplementary material for: Combinatorial Wnt signaling landscape during brachiopod anteroposterior patterning
Source: BMC Biol. 2024 Sep 19;22:212. doi: 10.1186/s12915-024-01988-w (PMC11414264; doi:10.1186/s12915-024-01988-w)
Supplement: Supplementary file 25 — Additional file 25: Table S1: [PDF] Gene accession numbers and primer pairs used for cloning the Wnt signaling components of the brachiopod Terebratalia transversa. [file 12915_2024_1988_MOESM25_ESM.pdf]

Gene	Accession	Forward	Reverse	Product
Ttra wnt1	KT253961	TAGCACACACAGGCAAGATAGTCC	GGAGTAGCAAGTGGAAATGGGG	935 bp
Ttra wnt1t	PP860497	AAGTTGAAGGCACGATGGACG	AGCATTCGCACAGACAGTAGCAC	1295 bp
Ttra wnt2	PP860498	CGAGTGTCAATGGCAGTTTAGGC	TGGAGGTTTTGTTGGGTTAGGAC	859 bp
Ttra wnt4	PP860499	ATGAACCTACACAACAACGA	ACAACACCAATGAAATTTACA	447 bp
Ttra wnt5	PP860500	TGGATAGGCGTAACAAAGAAGAGG	GATCTAGTCGGCTTCCTCAAACG	923 bp
Ttra wnt6	PP860501	ACCTCGTTTCGCATTGATTGAC	CCATCGTTTGAACCAAGCACC	990 bp
Ttra wnt7	PP860502	AATGAAGCAGGAAGGAGGGCAG	GCTGGGCATAAAATGTTGTGACG	947 bp
Ttra wnt8	PP860503	ATGAAGTGGGAAGAAGGGCA	GCAGCACCACTGGAATTTACA	458 bp
Ttra wnt9	PP860504	CGACTTTCTCAGATGGAATGCGAG	GTGAACGAACCAATCAGGATGC	988 bp
Ttra wnt10	PP860505	TACCCAACCGAAGAAGCAGACC	TACTTGATGCGTGACACCAGCG	1017 bp
Ttra wnt11	PP860506	TGGTTCCCATAAGTAGACAGAGACG	CACAATGCTGCCACAATCAAAG	897 bp
Ttra wnt16	PP860507	ATGTGTATTGGTCATGGGGCTC	GCATCGTGTGACAGTTTTCTCAAC	800 bp
Ttra wntA	PP860508	AGAGTAGGGAGACGGCATTCATC	TGTTTTCGGCAGTGTGGAGATAC	807 bp
Ttra fz1/2/7	PP860509	CGGAAAGGCTGTGAGGAAGT	GCCCAAGCAGCCAAATGAAA	1009 bp
Ttra fz4	PP860510	AGAGGAGTTTGGTGGCGAGG	AAGAGAAGGAACACAAGATTGGGTC	1247 bp
Ttra fz5/8	PP860511	CAAGAACTGAGTGAAGCCTATCCC	TGACCCACCAAAGAGATGATGC	1328 bp
Ttra fz9/10	PP860512	CCAATGTAAATGACAAGGGTAGCG	CAGCCAATAGAAAGACAGTGCCTG	902 bp
Ttra sfrp1/2/5	PP860513	CCACTTGTCCACAGTTGTCAGTCG	TTCGCAATCCTTCTTCACCG	884 bp
Ttra dkk5	PP860514	CAAGCGATCATTCGGTCAG	CCTTGTGAAAATATGTGGCTTC	762 bp
Ttra wif	PP860515	TTCAAAAACAAGGGCTTCTT	TGGTCTCCCAAACATTCACA	953 bp
Ttra dsh	PP860516	ATCCACTGCAATGTCATCCA	AGGGGAGCTAATGTATCCCT	951 bp
Ttra dgo	PP860517	GTGGATAGACAAGGCCAGGT	TAGATTCTCATTGTCTGTGT	972 bp
Ttra pk	PP860518	AGCCTTGTATGTCGTGTGGA	AAATGCTTCTGTTGCCATTC	949 bp
Ttra fmi	PP860519	ATTCTACAGGTGGTGCTGCT	TGATCTCCGTCAGCATACGG	951 bp
Ttra stbm	PP860520	TCTTGTTTCTGGTGTTTGTG	TTGCTATGTCTCCCTGTCGT	897 bp
Ttra jnk	PP860521	AAAACGGGAGGTCATTGCAC	GATCCTGGAAAGAGCACATT	952 bp
