# Supplementary material for: Combinatorial Wnt signaling landscape during brachiopod anteroposterior patterning
Source: BMC Biol. 2024 Sep 19;22:212. doi: 10.1186/s12915-024-01988-w (PMC11414264; doi:10.1186/s12915-024-01988-w)
Supplement: Supplementary file 6 — Additional file 6: Fig. S6: [PDF] Schematic drawings summarizing the expression of wnt1, wnt1t, wnt2, wnt4, wnt5, wnt6, and wnt7 in Terebratalia transversa. For each developmental stage of each gene, a blastoporal/ventral view (top) and a lateral view (bottom) are shown. Faded colors represent expression domains in the mesoderm or endoderm, or in the ectoderm when it is beneath the mantle lobe. [file 12915_2024_1988_MOESM6_ESM.pdf]

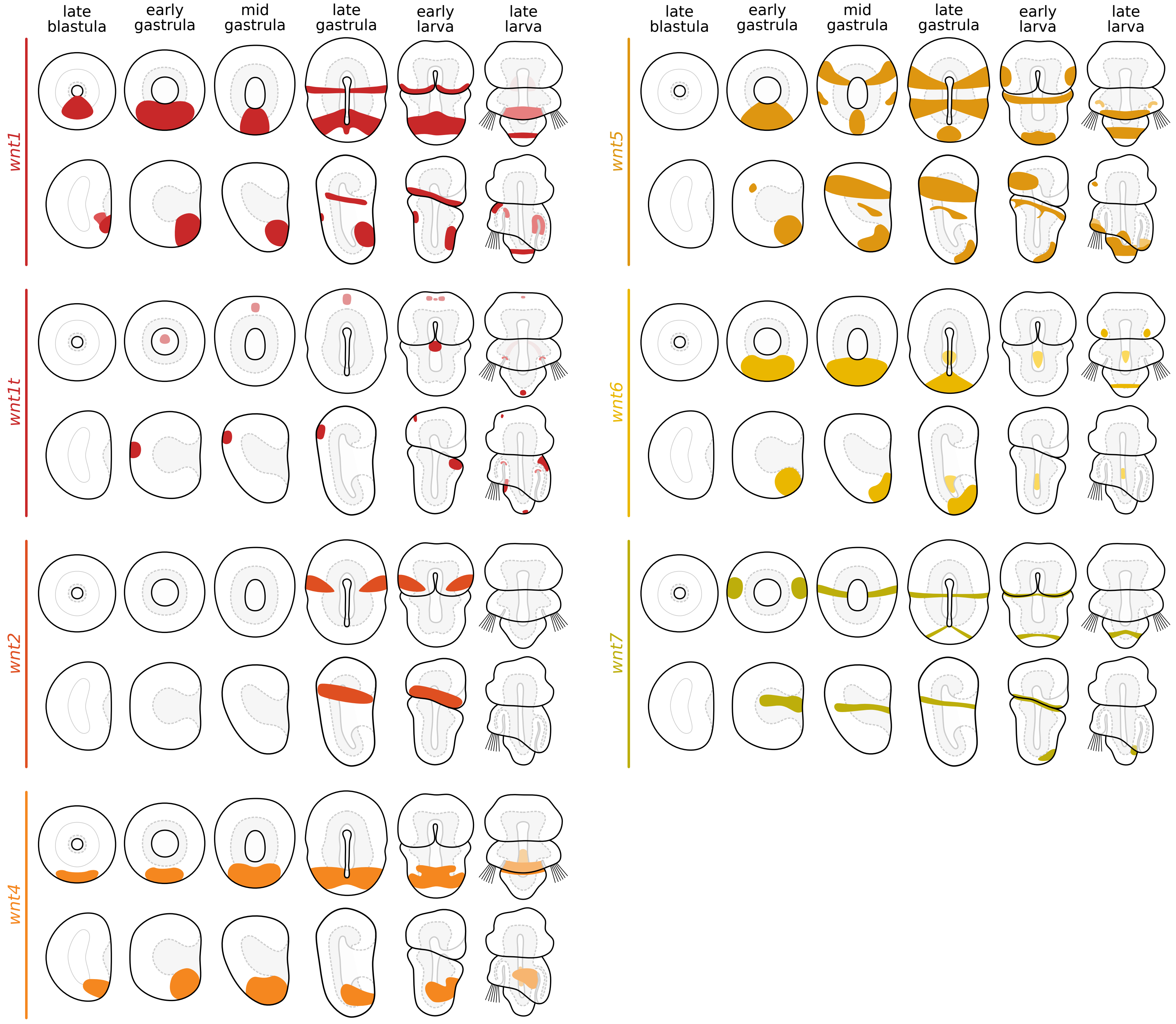
late blastula
early gastrula
mid gastrula
late gastrula
early larva
late larva
late blastula
early gastrula
mid gastrula
late gastrula
early larva
late larva
wnt1
wnt5
wnt1t
wnt6
wnt2
wnt7
wnt4
